# A systematic review and meta-analysis of the effects of antibiotic consumption on antibiotic resistance

**DOI:** 10.1186/1471-2334-14-13

**Published:** 2014-01-09

**Authors:** Brian G Bell, Francois Schellevis, Ellen Stobberingh, Herman Goossens, Mike Pringle

**Affiliations:** 1 Division of Primary Care, University of Nottingham, University Park, Nottingham NG7 2RD, UK; 2 NIVEL (Netherlands Institute for Health Services Research), PO Box 1568, 3500 BN Utrecht, the Netherlands; 3 Department of General Practice and Elderly Care Medicine/EMGO Institute for Health and Care Research, VU University Medical Centre, Amsterdam, the Netherlands; 4 Caphri University of Maastricht/Maastricht, University Hospital, Medical Microbiology, P.Debyelaan 25, 6229 HX Maastricht, the Netherlands; 5 University of Antwerpen, Universitair Ziekenhuis Antwerpen, Laboratory of Medical Microbiology, Wilrijkstraat 10, B-2650 Edegem, Belgium

**Keywords:** Antibiotic resistance, Antibiotic usage, Community-acquired infections, Meta-analysis

## Abstract

**Background:**

Greater use of antibiotics during the past 50 years has exerted selective pressure on susceptible bacteria and may have favoured the survival of resistant strains. Existing information on antibiotic resistance patterns from pathogens circulating among community-based patients is substantially less than from hospitalized patients on whom guidelines are often based. We therefore chose to assess the relationship between the antibiotic resistance pattern of bacteria circulating in the community and the consumption of antibiotics in the community.

**Methods:**

Both gray literature and published scientific literature in English and other European languages was examined. Multiple regression analysis was used to analyse whether studies found a positive relationship between antibiotic consumption and resistance. A subsequent meta-analysis and meta-regression was conducted for studies for which a common effect size measure (odds ratio) could be calculated.

**Results:**

Electronic searches identified 974 studies but only 243 studies were considered eligible for inclusion by the two independent reviewers who extracted the data. A binomial test revealed a positive relationship between antibiotic consumption and resistance (p < .001) but multiple regression modelling did not produce any significant predictors of study outcome. The meta-analysis generated a significant pooled odds ratio of 2.3 (95% confidence interval 2.2 to 2.5) with a meta-regression producing several significant predictors (F(10,77) = 5.82, p < .01). Countries in southern Europe produced a stronger link between consumption and resistance than other regions.

**Conclusions:**

Using a large set of studies we found that antibiotic consumption is associated with the development of antibiotic resistance. A subsequent meta-analysis, with a subsample of the studies, generated several significant predictors. Countries in southern Europe produced a stronger link between consumption and resistance than other regions so efforts at reducing antibiotic consumption may need to be strengthened in this area. Increased consumption of antibiotics may not only produce greater resistance at the individual patient level but may also produce greater resistance at the community, country, and regional levels, which can harm individual patients.

## Background

In the absence of the development of new generations of antibiotic drugs, appropriate use of existing antibiotics is needed to ensure the long term availability of effective treatment for bacterial infections [1]. If antibiotics become ineffective, then established and newly emerging infectious diseases, which are becoming an increasing threat, may lead to increased morbidity, health care utilisation and premature mortality [2-4].

Unfortunately, greater use of antibiotics during the past 50 years has exerted selective pressure on susceptible bacteria and may have favoured the survival of resistant strains [5], some of which are resistant to more than one antibiotic. If excessive antibiotic use can be reduced, the expectation is that resistant bacteria may be replaced by susceptible bacteria because resistant bacteria may be less ‘fit’ than susceptible bacteria [6].

More than 90% of antibiotics for medical use in Europe are prescribed to non-hospitalized patients [7]. However, existing information on antibiotic resistance patterns from pathogens circulating among community-based patients is substantially less than from hospitalized patients on whom guidelines are often based. We therefore chose to assess the relationship between the antibiotic resistance pattern of bacteria circulating in the community and the consumption of antibiotics in the community. Although Costelloe [8] et al. studied the relationship between consumption and resistance in primary care, they examined studies at the individual patient level only, omitting ecological studies (those conducted at the supra-individual level) which are included in this review. In this paper, we present a systematic review and meta-analysis of the literature on the relationship between antibiotic consumption in outpatient settings and antibiotic resistance of pathogens circulating in the community.

## Methods

### Search strategy

We searched both the English and non-English language literature for studies that looked at the relationship between antibiotic resistance and human antibiotic consumption in the community. An attempt was made to find the grey literature and published scientific literature in English, Spanish, French, German, Hungarian, Dutch, Swedish, and Croatian. These languages were chosen because the corresponding European countries were involved in the current project (Appropriateness of Prescribing Antibiotics in Primary Health Care with respect to Antibiotic Resistance). Search engines, such as Google Scholar, Embase, and Medline, were used to find published literature with reference lists of relevant articles searched by hand. Medline was searched from 1950 to late 2010 while Embase was searched from 1980 to late 2010. A list of search terms and connectors, which were used for both the English and non-English searches, can be found in Appendix 1.

### Study selection

We selected studies where a) the bacteria were acquired, and antibiotic consumption was measured, in the community b) the majority of the participants did not have a serious illness such as HIV or cancer c) the interval between consumption and resistance was one month or greater because we wanted to examine whether there was an enduring association between consumption and resistance and d) a statistical link between consumption and resistance was tested. Studies that presented descriptive information and made no attempt to establish a statistical connection between consumption and resistance were not included in the analysis. No limits were placed on when the study was published and few restrictions were placed on the type of study methodology, so both observational and experimental studies were included.

The level of analysis for any given study ranged from the individual patient (or individual bacterial isolate) level to the country (or groups of countries) level. Studies examined either children or adults or both, there were no restrictions on which body sites were sampled for establishing bacterial resistance or how antibiotic consumption was measured, and all bacteria and all antibiotics were considered relevant. Studies undertaken anywhere in the world were included with both English and non-English articles retrieved for our review. The list of the criteria we used to exclude studies is provided in Appendix 2.

To minimise the tendency to include studies that only reported significant results, we systematically searched the grey literature and included studies in which the primary focus of the article was not on the relationship between antibiotic consumption and antibiotic resistance. Abstracts were examined with full articles obtained and translated if the study looked relevant. When questions arose in applying the exclusion criteria, the authors resolved any disagreements through discussion.

### Data extraction

Full articles were examined for quality and data were independently extracted by the authors using purpose-built forms that listed the relevant variables. Any disagreements were resolved by discussing the articles and reaching consensus. Explanatory variables that were extracted from the studies are listed in Table 1.

**Table 1:** Explanatory variables

**Variable**	**Number (percentages in parenthesis) total sample = 243**	
Outcome	Positive	164 (67%)
	Negative or equivocal	79 (33%)
Level of sampling	Individual	72 (30%)
	Region/Country	124 (51%)
	Other	47 (19%)
Level of analysis	Individual	178 (73%)
	Region/Country	53 (22%)
	Other	12 (5%)
Children/Adults	Children	88 (36%)
	Adults	62 (26%)
	Both	93 (38%)
Bacteria*	*Streptococcus*	132 (54%)
	*Staphylococcus*	50 (21%)
	Enteric Bacteria	69 (28%)
	*Haemophilus*	24 (10%)
	Other	17 (7%)
Most common bacteria/Drug combinations**	*B*-lactam resistant *S pneumonia*	104 (43%)
	Macrolide resistant *S pneumonia*	56 (23%)
	Quinolone resistant *E coli*	41 (17%)
	*B*-lactam resistant *E coli*	35 (14%)
	Sulphonamide resistant *E coli*	31 (13%)
	Methicillin-resistant *S aureus*	38 (16%)
Most common antibiotics consumed***	*B*-lactams	132 (54%)
	Macrolides	93 (38%)
	Sulphonamides	59 (24%)
	Quinolones	52 (21%)
	Antibiotic not specified	65 (27%)
Time between consumption and resistance^	Six months or less	129 (53%)
	More than 6 months	57 (23%)
	Same time	43 (18%)
	Not specified	14 (6%)
How antibiotic consumption was assessed#	Self report	99 (41%)
	Medical records	92 (38%)
	Sales/Prescriptions	65 (27%)
	Direct application of antibiotic	14 (6%)
Region where study was conducted##	Northern Europe	66 (27%)
	Southern Europe	45 (18%)
	US	67 (28%)
	Other	61 (25%)
Type of study	Cross-sectional	101 (42%)
	Ecological	56 (23%)
	Case–control	35 (14%)
	Quasi-experiment	21 (9%)
	Other	30 (12%)

*Percentages do not equal 100% as any given study may have examined more than one type of bacteria.

**Percentage do not equal 100% as any given study may have examined more than one combination; resistant and non-susceptible strains of *S pneumonia* are combined under the resistant label for this bacterium.

***Percentages do not equal 100% as any given study may have examined more than one antibiotic.

^This variable represents the maximum time interval in any given study between when consumption occurred and resistance was measured. Studies classified as ‘same time’ tended to be ecological studies where the precise interval separating consumption and resistance could not be determined, these studies often simply reported consumption and resistance occurring together over some multi-year interval.

#Percentages do not equal 100% as any given study may have used more than one method.

##The total equals 239 as three studies were conducted in both southern Europe and northern Europe and one study was coded as not applicable.

The main dependent variable was a dichotomous coding of study outcome based on whether or not the article supported a positive relationship between antibiotic consumption and antibiotic resistance. A positive relationship could be represented by either increased consumption associated with increased resistance or decreased consumption associated with decreased resistance. A negative relationship could be represented by either the absence of a significant relationship between consumption and resistance or, in rare instances, a truly negative relationship, such as increased consumption associated with decreased resistance. Studies that did not clearly provide either positive or negative evidence were combined with studies that produced a negative relationship to form a not-positive category which was compared with the positive studies in the data analysis. Studies were classified as positive or negative based on a preponderance of the evidence with a positive outcome recorded when an increase in antibiotic consumption was associated with either an increase in resistant bacteria or a decrease in susceptible bacteria. For the meta-analysis the dependent variable was the odds ratio for the study based on the standardized effect size.

### Data analysis

We wanted to use all of the studies in our analysis in order to improve power and examine whether a relationship between antibiotic consumption and antibiotic resistance existed for the complete set of data, so we first ran simple correlations and a logistic regression because the data did not generally lend themselves to a meta-analytic approach. The studies were heterogeneous with study design and effect size measures varying greatly between studies. Furthermore, a single effect size could not be calculated for many studies because numerous associations between consumption and resistance were reported and simple averaging of effect sizes for any given study was not appropriate. For those studies (N = 88) for which a single effect size measure could be obtained a meta-analysis and meta-regression analysis were conducted.

We ran a series of correlations between each of the explanatory variables and the dichotomous outcome variable in order to reduce the number of predictors in the subsequent regression and ensure that the number of cases (studies) relative to the number of predictors was adequate. Variables that were significantly correlated at this level were used in a logistic regression and entered in a single step to predict whether a positive or negative relationship was found between consumption and resistance. A binomial test was also run to see whether a significantly greater number of positive associations between consumption and resistance were found in the studies. The binomial test examined whether the proportion of studies with a positive association between consumption and resistance significantly differed from the proportion of studies with a positive association that would be expected under the null hypothesis. The proportion under the null hypothesis (no association between resistance and consumption) was 50% (i.e., the probability of a positive association was equal to the probability of a non-positive association). For a subset of the studies, a meta-analysis and subsequent meta-regression analysis were run. Analyses were conducted in SPSS version 16 and Stata version 11.2.

## Results

### Application of exclusion criteria

**Figure 1:**
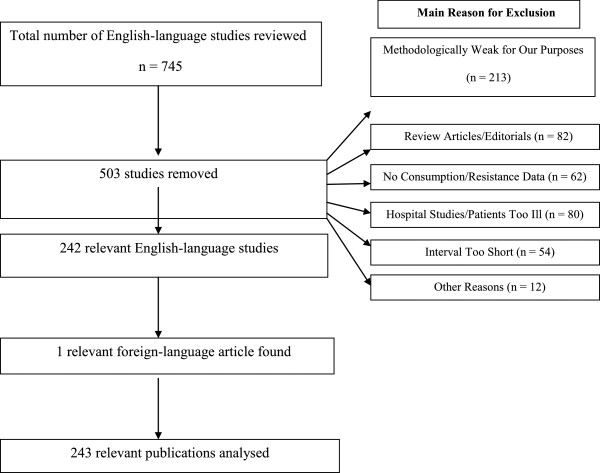
How studies were selected.

To arrive at the studies that formed the basis of our analysis (see Figure 1), we searched the English language literature and identified 745 studies that appeared relevant. However, 503 studies (68%) did not meet our inclusion criteria. Most of the excluded studies (over 95%) were eliminated for one of the following five reasons: methodological weaknesses (N = 213) such as no attempt to statistically analyse the data, review article or editorial (N = 82), study did not measure either resistance or consumption (N = 62), hospital study or the patients were too ill (N = 80), and short interval (less than a month) between the measurement of consumption and resistance (N = 54). Altogether 242 English-language studies or 32% (239 published studies and 3 grey literature documents) of the 745 originally identified studies were considered relevant. A list of excluded studies is available from the authors upon request.

Searches conducted by colleagues in other countries revealed 229 studies that appeared relevant, but only one of these studies was included in our analysis. Non-English articles were excluded, with three exceptions, because either a version of the study had already been obtained in English or the study did not provide statistical analysis of a link between antibiotic consumption and resistance in the community. The relevant non-English language article (Appendix 3: Dellamonica et al 2002) along with the 242 English language articles provided us with a total of 243 studies that formed the basis of our analysis.

### Study characteristics

Table 1 shows the number of studies that fell into various categories for the 243 studies included in the final analysis. More than two-thirds of the studies found a positive relationship between antibiotic resistance and antibiotic consumption. Most of the studies (51%) sampled data at the regional or country level but analysed data at the individual level (73%) which means that data from individual participants were often sampled from some larger unit, such as an entire country.

With few exceptions, the bacteria that were studied fell into one of three classes (*Streptococcus*, *Staphylococcus*, or Enteric bacteria, such as *E coli*). The most common classes of antibiotics for which consumption was measured included *B*-lactams, macrolides, sulphonamides, and quinolones although more than a quarter of the studies did not specify which antibiotics had been consumed.

The studies in our sample were usually conducted in either Europe or the US. More studies took place in northern Europe than in southern Europe although both regions were well represented. Almost 80% of the studies used one of three study designs: cross-sectional, ecological, or case–control.

### Predicting study outcome

We used the binomial test to examine whether there were more studies that produced a positive outcome in which either increased consumption was associated with increased resistance or decreased consumption associated with decreased resistance. The binomial test revealed that the probability of a positive outcome was significantly greater than the probability of an outcome that was not positive (p < .001).

We next examined the correlations between the predictor variables listed in Table 1 and our dichotomous outcome measure. Each of the categorical variables listed in Table 1 was turned into a set of dichotomous variables for this purpose. For example, the adult/child variable was recoded into three dichotomous variables, one representing children, another representing adults, and a third representing both children and adults.

Three positive correlations were found: studies that included both adults and children were more likely to find a positive association between antibiotic consumption and resistance (r = 0.17, p < .01) as were studies that examined tetracycline-resistant *S pneumonia* (r = 0.14, p < .05) and those that looked at quinolone consumption (r = 0.15, P < .05). Three negative correlations were also produced: for studies that only contained children (r = −0.13, p < .05), those conducted in the US (r = −0.16, p < .05) and cross-sectional studies (r = −0.15, p < .05). Studies which contained children, those conducted in the US, and cross-sectional studies were more likely to find a small, either negative or not positive, association between antibiotic consumption and antibiotic resistance.

All of the variables for which significant correlations were found were simultaneously entered into a logistic regression equation to predict study outcome. None of the variables that produced significant bivariate correlations were significant predictors in the logistic regression equation, although as a set, they did significantly predict the outcome variable (chi-square = 22.81, *df* = 6, p < .01) thereby distinguishing between studies with a positive association and other studies. However, only a little more than 10% of the variance in the outcome variable was explained by these predictors (Nagelkerke R^2^ = 0.12).

We noticed that the relationship between consumption and resistance often varied within a study depending on which bacteria were being considered. So, a study that looked at multiple types of bacteria may have found a positive relationship between consumption and resistance for one but a negative relationship for another. Our global outcome measure did not allow us to investigate these differences because it was based on the preponderance of evidence across bacterial classes for any given study. Therefore, we decided to conduct a separate analysis for each bacterial category (Streptococcus, Staphylococcus, Enteric Bacteria, and Haemophilus/Other). We recoded the outcome measure for those studies that contained more than one type of bacteria so that the outcome measure in any given analysis was based on the relationship between consumption and resistance for a single type of bacteria.

First, looking at the results from the binomial tests, we found that only enteric bacteria and streptococcus produced significantly more positive outcomes than negative outcomes (p < .01 for enteric bacteria and p < .001 for streptococcus). For Staphylococcus and Haemophilus/Other, the results from the binomial tests were not significant. Therefore, we decided to focus on enteric bacteria and streptococcus in subsequent analyses.

When correlations were run for the streptococcus bacteria, only two variables out of 48 were significantly correlated with outcome. Tetracycline resistant S pneumonia was significantly correlated with the outcome variable (r = 0.19, p < .05) and so was trimethoprim consumption (r = −0.23, p < .01). However, this is probably due to chance as the number of significant correlations was less than 5% of the total number of correlations that were run, so a logistic regression was not conducted. In a similar vein, only two correlations were significant for enteric bacteria. Patients who were children (r = −0.28, p < .05) and quinolone consumption (r = 0.33, p < .01) were significantly correlated with outcome. Again, with only two significant correlations, we can conclude that the results are probably due to chance, so a logistic regression was not run.

### Meta-analysis

We conducted a fixed-effects meta-analysis of a subset of studies for which a common effect size could be obtained. A fixed effects meta-analysis was more appropriate than a random effects meta-analysis because we wanted to determine whether heterogeneity between the studies could be explained by our predictors [9]. Our meta-analysis focused on the most common study designs and included articles that used cross-sectional, case–control, cohort, non-randomised quasi-experimental and randomized controlled trial designs. We ran a separate meta-analysis for each of the most common study designs (53 cross-sectional studies, 21 case–control studies, and 8 cohort studies) to see whether the results varied by study design. Ecological studies, which were conducted at the regional or country level, were excluded due to much larger sample sizes that would not allow comparisons with the much smaller studies that were used in this analysis. We decided against conducting a separate meta-analysis of the ecological studies because there were only 17 such studies for which a common effect size measure could be calculated. More studies than this would have been needed to explore relationships between predictors and effect size in any subsequent meta-regression. For the 88 studies that were included we were able to either use the odds ratio that was reported in the study or calculate one from the available data.

**Figure 2:**
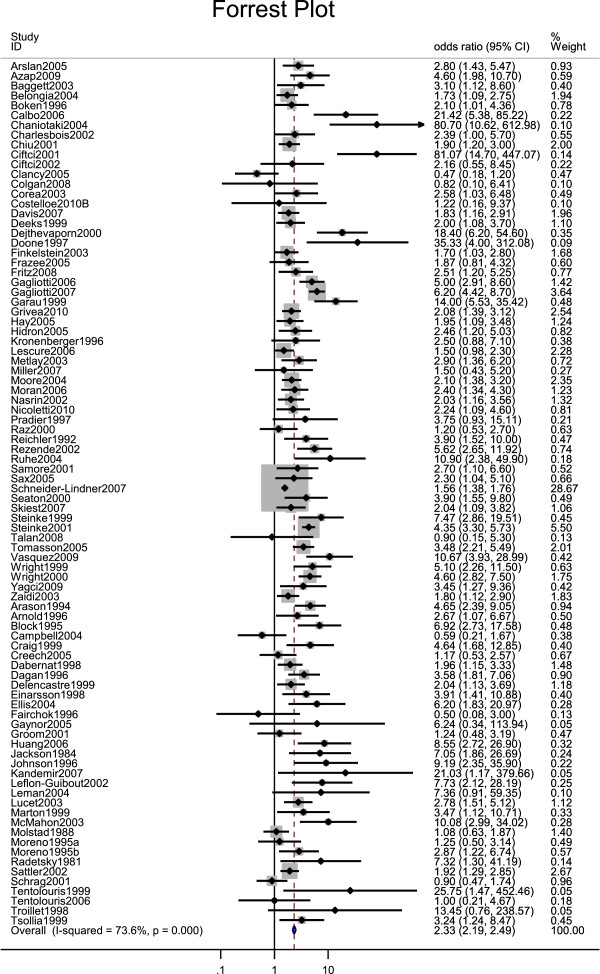
Forest plot showing odds ratio and 95% confidence interval for each study along with study weight.

Our meta-analysis revealed that there was a significant positive relationship between antibiotic consumption and resistance with a pooled effect size (odds ratio) of 2.33 (z = 25.71, p < .01, 95% confidence interval 2.19 to 2.49). The results were similar for each of the most common study designs including cross-sectional (OR = 2.46, z = 17.06, p < .01, 95% CI = 2.22 to 2.73), cohort (OR = 2.93, z = 7.02, p < .01, 95% CI = 2.17 to 3.96) and case–control studies (OR = 2.26, z = 17.41, p < .01, 95% CI = 2.07 to 2.48) so further analyses were conducted with all 88 studies. The forest plot in Figure 2 displays the odds ratio and weight for each study. One of the studies (Appendix 3: Schneider-Lindner et al 2007) was weighted more heavily than the other studies due to a very small standard error for the odds ratio but removing this study did not change our findings (OR = 2.74, z = 25.86, p < .01).

**Figure 3:**
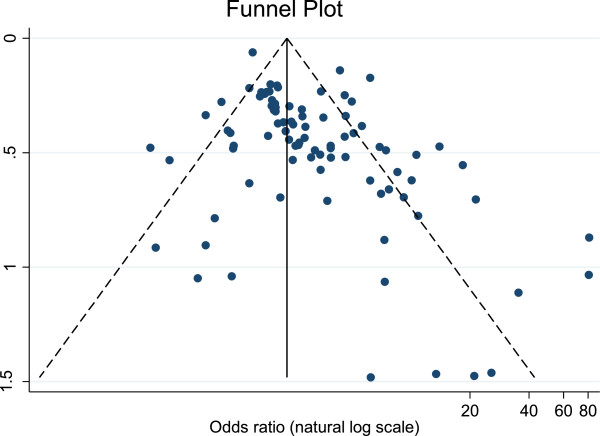
Funnel plot showing effect size (odds ratio) as a function of sample size (natural log of standard error).

We should also mention, as can be seen from the funnel plot in Figure 3, that there was some evidence of potential publication bias in our sample of studies. The absence of small studies in the lower left hand side of the plot suggests that small studies that did not find a strong association between antibiotic consumption and resistance may not have been published, although as Moller and Jennions [10] note, publication bias is not the only explanation for a skewed funnel plot.

Significant heterogeneity was observed with a heterogeneity chi-squared value of 329.75 (p < .01). The percentage of variation between studies due to heterogeneity was high (73.6%) so we decided to examine whether our independent variables could explain differences between studies in the odds ratios. Using weighted correlations, in which the correlation between the independent variable and the odds ratio was weighted by the corresponding standard error, we found 11 significant correlations (see Table 2) and then conducted a meta-regression to determine the independent effect of each variable on the odds ratio. We excluded the variable staphylococcus because it was highly correlated with Methicillin-Resistant *S aureus* (MRSA (r = 0.97)).

**Table 2:** Weighted correlations between odds ratios and independent variables

**Variable**	**Correlation**	**Significance**	Sample size*
Both children and adults	0.32	p < .01	20
Enteric bacteria	0.45	p < .01	20
*Staphylococcus*	−0.44	p < .01	30
MRSA	−0.48	p < .01	29
QREC	0.37	p < .01	10
*B*-lactam consumption	−0.31	p < .01	41
Sulphonamides consumption	−0.28	p < .01	20
Macrolide consumption	−0.24	p < .05	16
Quinolones consumption	−0.22	p < .05	12
Northern Europe	−0.22	p < .05	14
Southern Europe	0.24	p < .05	15

*Number of cases in each category out of 88 cases total.

The meta-regression showed that our set of independent variables significantly predicted the odds ratios (F (10, 77) = 5.82 p < .01) although significant residual variability remained (residual sum of squares = 187.78, *df* = 77, p< .01). Five variables were significant independent predictors: ‘both children and adults’ z = 3.90 < .01, ‘southern Europe’ z = 2.25 p < .05, ‘*B*-lactam consumption’ z = −3.15 p < .01, ‘MRSA’ z = −4.58 p < .01, and ‘quinolone-resistant *E coli’* (QREC) z = 2.57 p < .05. We can conclude that studies which contained both adults and children, those conducted in southern Europe, and studies that looked at QREC were more likely to find a strong positive relationship between antibiotic consumption and resistance. Studies that examined *B*-lactam consumption or MRSA tended to find a weaker relationship between consumption and resistance. The finding that southern European countries produced a much stronger link between resistance and consumption confirms previous observations that antibiotic resistance due to the consumption of antibiotics may be a greater problem in southern Europe than in northern Europe (Appendix 3: Goossens et al 2005).

### Publication bias

As noted earlier, there was some evidence of publication bias in the subsample of studies that formed our meta-analysis. Turning to the issue of publication bias for the full set of 243 studies, a large number of these studies (a third of our sample) did not find a positive association between antibiotic consumption and antibiotic resistance, which reduces the likelihood of publication bias and lessens concerns that we chose studies for inclusion simply because they supported our hypothesis. Concerns with publication bias are also reduced by our contention that studies which reported negative evidence probably would be published since studies that found no relationship between consumption and resistance would still generate a great deal of interest. To examine potential publication bias further, we looked at 92 studies that were primarily concerned with something other than the relationship between consumption and resistance. These studies, which should be less prone to the publication bias that we are concerned with, found a roughly similar split in the ratio of positive to not positive outcomes that we found with the larger sample, 60% of the studies found a positive relationship and 40% did not find a positive association.

## Discussion

Our initial analysis with the entire sample of 243 studies revealed that there was a positive association between bacterial resistance and antibiotic consumption in the community, which means that either increased consumption was associated with increased resistance or decreased consumption was associated with decreased resistance. Using a much larger sample of studies we found support for Costelloe’s [8] conclusion that antibiotic prescribing is associated with the development of antibiotic resistance. We also extended Costelloe’s work by including studies that were conducted at both the ecological level and the individual level and finding that level of analysis did not affect the relationship between consumption and resistance.

Although several study variables were significantly correlated with study outcome (whether or not a positive association was obtained between resistance and consumption), all of the correlation coefficients were small (0.20 or less), and none of these variables were significant predictors of study outcome when they were entered into a logistic regression equation. A lack of significant predictors means that the association between antibiotic consumption and bacterial resistance does not depend on any of the demographic variables that we investigated or on other variables of interest, such as the level at which the data were sampled or analysed. Although no significant predictors were found when individual classes of bacteria were examined either, the binomial test showed that the positive relationship between consumption and resistance was only obtained for enteric bacteria and streptococcus so efforts at controlling resistance needs to focus on these bacteria.

When the 88 studies in our meta-analysis were examined, we found that there was a significant positive relationship between consumption and resistance and several variables that were significant predictors. Studies that contained both adults and children, those conducted in southern Europe, and studies that looked at QREC were more likely to find a strong positive relationship between antibiotic consumption and resistance whereas studies that examined *B*-lactam consumption or MRSA tended to find a weaker relationship between consumption and resistance. The finding that southern European countries produced a much stronger link between resistance and consumption confirms previous observations that antibiotic resistance due to the consumption of antibiotics may be a greater problem in southern Europe than in northern Europe (Appendix 3: Goossens et al 2005). Also, the results from the meta-analysis indicate that the use of quinolones may need to be reduced when treating *E Coli* infections.

One of the major strengths of our review is the large number of studies that contributed to our analysis, which means that we had plenty of power to detect an effect. We placed few restrictions on the types of studies that we considered relevant, which make our findings applicable to a wide range of settings thereby increasing ecological validity and also reducing publication bias in which non-published studies that do not produce significant findings are overlooked. Our review included English-language studies and studies published in other languages, observational and experimental studies, grey literature as well as published scientific literature, with no geographical restrictions placed on where the study was conducted. For any given study the level of analysis ranged from the individual patient (or individual isolate) level to the country (or groups of countries) level and both children and adults were well represented. There were no restrictions on which body sites were sampled for establishing bacterial resistance, how antibiotic consumption was measured, which bacteria were isolated, or which antibiotics were consumed. In spite of the comprehensive nature of our review, a final strength is our targeted approach to answering the primary question, is there a relationship between antibiotic consumption and antibiotic resistance in the community, by the use of a clear set of exclusion criteria.

Potential limitations should also be noted with our review. First, our primary outcome measure was based on a dichotomous coding of whether or not a study supported a positive relationship between antibiotic consumption and antibiotic resistance so it ignored possible differences within studies because it was based on the preponderance of evidence for any given study. However, we believe that using a global measure of outcome to represent the relationship between antibiotic consumption and resistance was justified as a common effect size measure could not be calculated for most of the studies that were used in our review. Another limitation concerns poor reporting of important information and the use of weak measures in many studies. More than a quarter of the included studies did not specify which antibiotic had been consumed and almost half (41%) of the studies used self-reports, which may be unreliable, to assess antibiotic consumption. The most serious limitation in this regard concerned inadequate reporting of how much time had elapsed between antibiotic consumption and the measurement of bacterial resistance. Most studies did not provide the precise interval between when antibiotics were consumed and when resistance was measured, which hindered our attempts to determine whether resistance endured over time as Costelloe [8] had done. A final limitation concerns some evidence of potential publication bias in our meta-analysis in which smaller studies that did not find a strong positive association between antibiotic consumption and resistance may not have been published.

## Conclusions

To conclude, our literature review reveals the following. First, there is an association between antibiotic consumption and the subsequent development of bacterial resistance at both the individual and community level. For clinicians this is important because our findings do not just apply at the individual patient level but also at the community, country and regional levels. As Bergman (Appendix 3: Bergman et al 2006) noted, antibiotic pressure at the population level may be more important than the individual’s use of antibiotics in determining that individual’s risk of harbouring resistant bacteria. Both responsible prescribing at the individual level as well as public policy that addresses the problem at the national or regional level are critical components of any strategy to reduce bacterial resistance, which supports the current efforts of many countries, including the UK, to ensure that antibiotics are only used when indicated and that the most appropriate antibiotic (often an older established antibiotic) is used. We also found that the link between antibiotic consumption and resistance does not depend on any of the demographic variables that we investigated which means that antibiotic consumption may lead to resistance for diverse groups of people in various settings, although we would hasten to add that several important predictors were identified in the meta-analysis including a stronger link between resistance and consumption in southern Europe than in other regions. This discrepancy may have been due to the outcome measure that was used or the particular mix of studies that was examined in the meta-analysis. More work needs to be done because significant residual variability remained that could not be accounted for by the variables that were used in our meta-analysis.

Future work should also address the issue of co-selection in which the use of one antibiotic produces resistance to another antibiotic. If co-selection is widespread, then resistance to one antibiotic could be due to the use of another antibiotic that was not measured in the study under investigation, in which case the conclusion that there was no association between consumption and resistance would be misleading. Unfortunately, the studies that we examined rarely looked at this issue. More thought also needs to be given to improving measures of antibiotic consumption. Proxy measures, such as patient self-report of antibiotic use, do not directly assess consumption and therefore may be of limited utility.

## Appendix 1 Search terms and connectors (AND/OR) for literature search

‘Drug Resistance, Microbial’

OR

‘Drug Resistance, Bacterial’

OR

‘Bacterial Resistan*’ (which captures terms such as ‘Resistant’ and ‘Resistance’)

OR

‘Antimicrobial Resistan*’

OR

‘Antibiotic Resistan*’

AND

‘Consumption’

OR

‘Antibiotic Consumption’

OR

‘Antibiotic Prescri*’ which includes terms such as ‘Prescribing’ and ‘Prescription’

OR

‘Antibiotic Utilization’

OR

Antibiotic Use’

OR

‘Antibiotic Sales’

AND

‘Community’

OR

‘Primary Care’

OR

‘Primary Health Care’

OR

‘General Practice’

OR

‘Family Practice’

OR

‘Ambulatory Care’

## Appendix 2 Exclusion criteria

A). Some studies were excluded because the infection was not community acquired or the setting was not appropriate:

1) Studies where more than half of the resistant bacteria were acquired in a hospital setting or in another institutional setting such as a nursing home.

1) Studies where antibiotic consumption was only measured in a hospital or nursing home, not in the community

1) In vitro studies unless in vivo resistance was also examined

1) Studies that examined the topical use of antibiotics for skin infections, dental use of antibiotics, veterinary studies, and those that looked at the use of antibiotics in agriculture or as antiseptics

1) Studies that looked at the effects of bacteria from waste or industry on soil, air and water

1) Studies that looked at viruses, parasites or fungi

1) Studies that only looked at treatment failure or eradication of bacteria unless resistance was also measured

1) Studies where most of the patients were seriously ill (such as, when over half the patients were on immunosuppressive therapy, were HIV positive, had meningitis or end-stage cancer, were using catheters, or were recruited in tertiary centres). Those studies that looked at urinary tract infection were only included when at least 2/3 of the sample contained women (not girls) with an uncomplicated urinary tract infection.

B) Some studies conducted at the level of the individual patient (as opposed to country or region) were excluded because the temporal relationship between consumption and resistance was not clear or the interval between consumption and resistance was too short (we wanted evidence of an enduring effect):

9) Studies in which consumption was measured after resistance or where the time between consumption and resistance was exclusively less than one month. Patients could be currently taking antibiotics at the time that resistance was measured if the long term use of antibiotics was investigated (such as the effect of antibiotics received in the past 6 months on resistance to current treatment with antibiotics)

C) Some studies were given less weight than others:

10) We focused on newer studies (those conducted since 1990) although some older studies from the 1970s and 1980s were also included. [Older studies tended to be methodologically inferior (in reporting of statistics) and resistance tends to be greater in newer studies, which is an important consideration as the current relationship between resistance and consumption is more important than the relationship from 40 or 50 years ago].

10) Review articles were not included (except as a source of references) unless the study combined previous work in a meta-analysis or systematic review.

10) Studies that simply looked at the biological mechanisms which produced resistance were excluded (However, some studies examined bacteria that produced resistance in a particular way, such as b-lactamase-producing bacteria. These studies were included because they looked at the effects of antibiotic consumption on the production of a particular type of resistant bacteria).

10) Studies that looked at treatment failure or bacterial eradication were excluded unless resistance was also measured. For example, some studies examined C Difficile, which is difficult to eradicate, but these articles were excluded unless resistance was also measured.

D) Studies were generally classified as weak, and therefore excluded, when:

14) The authors acknowledged that there was insufficient power to detect an effect

14) No statistical analysis was conducted. In some cases, conclusions were based on graphs and figures with no statistical results provided, for other studies a single participant or a handful of participants was studied so no statistical tests were conducted.

14) Poor measures were used, usually poor consumption measures (such as relying on participant self-report when recall was poor).

## Appendix 3 Included studies

1. Albrich, W.C., Monnett, D.L., & Harbarth, S., *Antibiotic selection pressure and resistance in Streptococcus pneumoniae and Streptococcus pyogenes.* Emerging Infectious Diseases, 2004. **10**(3): p. 514–517.

2. Alos, J.I., Serrano, M.G., Gomez-Garces, J.L., & Perianes, J., *Antibiotic resistance of Escherichia coli from community-acquired urinary tract infections in relation to demographic and clinical data.* Clinical Microbiology and Infection, 2005. **11**(3): p. 199–203.

3. Arason, V.A., Kristinsson, K.G., Sigurdsson, J.A., Gudmundsson, S., Molstad, S., & Dottir, G.S., *The effect of antimicrobial (AM) use on carriage of pencillin resistant pneumococci (PRP)*, in *34th Interscience Conference on Antimicrobial Agents and Chemotherapy*. 1994, American Society for Microbiology: Orlando, FL USA. p. 85 Abstract C.

4. Arason, V.A., Kristinsson, K., Sigurdsson, J., Stefansdottir, G., Molstad, J., & Gudmundsson, S., *Do antimicrobials increase the carriage rate of penicillin resistant pneumococci in children? Cross sectional prevalence study.* British Medial Journal, 1996. **313**: p. 387–391.

5. Arason, V.A., Gunnlaugsson, A., Sigurdsson, J.A., Erlendsdottir, H., Gudmundsson, S., & Kristinsson, K.G., *Clonal spread of resistant pneumococci despite diminished antimicrobial use.* Microbial Drug Resistance, 2002. **8**(3): p. 187–192.

6. Arason, V.A., Sigurdsson, J.A., Erlendsdottir, H., Gudmundsson, S., & Kristinsson, K.G., *The role of antimicrobial use in the epidemiology of resistant penumococci: A 10-year follow up.* Microbial Drug Resistance, 2006. **12**(3): p. 169–176.

7. Arnold, K.E., Leggiadro, R.J., Breiman, R.F., Lipman, H.B., Schwartz, B., Appleton, M.A., Cleveland, K.O., Szeto, H.C., Hill, B.C., Tenover, F.C., Elliott, J.A., & Facklam, R.R., *Risk factors for carriage of drug-resistant Streptococcus pneumoniae among children in Memphis, Tennessee.* The Journal of Pediatrics, 1996. **128**(6): p. 757–764.

8. Arslan, H., Azap, O.K., Ergonul, O., & Timurkaynak, F. on behalf of the Urinary Tract Study Group, *Risk factors for ciprofloxacin resistance among Escherichia coli strains isolated from community-acquired urinary tract infections in Turkey* Journal of Antimicrobial Chemotherapy, 2005. **56**(5): p. 914–918.

9. Azap, O.K., Arslan, H., Serefhanoglu, K., Colakoglu, S., Erdogan, H., Timurkaynak, F., & Senger, S.S., *Risk factors for extended-spectrum B-lactamase positivity in uropathogenic Escherichia coli isolated from community-acquired urinary tract infections.* Clinical Microbiology and Infection, 2009. **16**(2): p. 147–151.

10. Baggett, H.C., Hennessy, T.W., Leman, R., Hamlin, C., Bruden, D., Reasonover, A., Martinez, P., & Butler, J.C., *An outbreak of community-onset methicillin-resistant Staphylococcus aureus skin infections in southwestern Alaska.* Infection Control and Hospital Epidemiology, 2003. **24**(6): p. 397–402.

11. Baggett, H.C., Hennessy, T.W., Rudolph, K., Bruden, D., Reasonover, A., Parkinson, A., Sparks, R., Donlan, R.M., Martinez, P., Mongkolrattanothai, K., & Butler, J.C., *Community-onset methicillin-resistant Staphylococcus aureus associated with antibiotic use and the cytotoxin Panton-Valentine leukocidin during a furunculosis outbreak in rural Alaska.* Journal of Infectious Diseases, 2004. **189**(9): p. 1565–1573.

12. Bartoloni, A., Cutts, F., Leoni, S., Austin, C.C., Mantella, A., Guglielmetti, P., Roselli, M., Salazar, E., & Paradisi, F., *Patterns of antimicrobial use and antimicrobial resistance among healthy children in Bolivia.* Tropical Medicine and International Health, 1998. **3**(2): p. 116–123.

13. Bassetti, M., Mantero, E., Gatti, G., Di Biagio, A., & Bassetti, D., *Streptococcus pyogenes erythromycin resistance in Italy.* Emerging Infectious Diseases, 1999. **5**(2): p. 302–303.

14. Beekman, S.E., Diekema, D.J., Heilmann, K.P., Richter, S.S., & Doern, G.V., *Macrolide use identified as risk factor for macrolide-resistant Streptococcus pneumoniae in a 17-center case–control study.* European Journal of Clinical Microbiology and Infectious Diseases, 2006. **25**(5): p. 335–339.

15. Belongia, E.A., Sullivan, B.J., Chyou, P., Madagame, E., Reed, K.D., & Schwartz, B., *A community intervention trial to promote judicious antibiotic use and reduce pencillin-resistant Streptococcus pneumoniae carriage in children* Pediatrics, 2001. **108**(3): p. 575–583.

16. Ben-Ami, R., Rodriguez- Bano, J., Arslan, H., Pitout, J.D.D., Quentin, C., Calbo, E.S., Azap, O.K., Aroin, C., Pascual, A., Livermore, D.M., Garau, J., & Carmeli, Y., *A multinational survey of risk factors for infection with extended-spectrum B-lactamase-producing enterobacteriaceae in nonhospitalized patients.* Clinical Infectious Diseases, 2009. **49**(5): p. 682–690.

17. Bergman, M., Huikko, S., Pihlajamaki, M., Laippala, P., Palva, E., Huovinen, P., Seppala, H. & the Finnish Study Group for Antimicrobial Resistance, *Effect of macrolide consumption on erythromycin resistance in Streptococcus pyogenes in Finland in 1997–2001.* Clinical Infectious Diseases, 2004. **38**(9): p. 1251–1256.

18. Bergman, M., Huikko, S., Huovinen, P., Paakkari, P., Seppala, H. & the Finnish Study Group for Antimicrobial Resistance (FiRe Network), *Macrolide and azithromycin use are linked to increased macrolide resistance in Streptococcus pneumoniae.* Antimicrobial Agents and Chemotherapy, 2006. **50**(11): p. 3646–3650.

19. Bergman, M., Nyberg, S.T., Huovinen, P., Paakkari, P., Hakanen, A.J., & the Finnish Study Group for Antimicrobial Resistance, *Association between antimicrobial consumption and resistance in Escherichia coli.* Antimicrobial Agents and Chemotherapy, 2009. **53**(3): p. 912–917.

20. Bischoff, W.E., Wallis, M.L., Tucker, K.B., Reboussin, B.A., & Sherertz, R.J., *Staphylococcus aureus nasal carriage in a student community: Prevalence, clonal relationships, and risk factors.* Infection Control and Hospital Epidemiology, 2004. **25**(6): p. 485–491.

21. Blaettler, L., Mertz, D., Frei, R., Elzi, L., Widmer, A.F., Battegay, M., & Fluckiger, U., *Secular trend and risk factors for antimicrobial resistance in Escherichia coli isolates in Switzerland 1997–2007.* Infection, 2009. **37**(6): p. 534–539.

22. Block, S.L., Harrison, C.J., Hedrick, J.A., Tyler, R.D., Smith, R.A., Keegan, E., & Chartrand, S.A., *Pencillin-resistant Streptococcus pneumoniae in acute otitis media: risk factors, susceptibility patterns and antimicrobial management.* The Pediatric Infectious Disease Journal, 1995. **14**(3): p. 751–759.

23. Boccia, D., Alegiani, S.S., Pantosti, A., Moro, M.L., & Traversa, G., *The geographic relationship between the use of antimicrobial drugs and the pattern of resistance for Streptococcus pneumoniae in Italy.* European Journal of Clinical Pharmacology, 2004. **60**(2): p. 115–120.

24. Boken, D.J., Chartrand, S.A., Goering, R.R.V., Kruger, R., & Harrison, C.J., *Colonization with pencillin-resistant Streptococcus pneumoniae in a child-care center.* Pediatric Infectious Disease Journal, 1995. **14**(10): p. 879–884.

25. Boken, D.J., Chartrand, S.A., Moland, E.S., & Goering, R.V., *Colonization with pencillin-nonsusceptible Streptococcus-pneumoniae in urban and rural child-care centers.* The Pediatric Infectious Disease Journal, 1996. **15**(8): p. 667–672.

26. Borgmann, S., Jakobiak, T., Gruber, H., Schroder, H., & Sagel, U., *Prescriptions of broad-spectrum antibiotics to outpatients do not match increased prevalence and antibiotic resistance of respiratory pathogens in Bavaria.* Polish Journal of Microbiology, 2009. **58**(2): p. 105–110.

27. Borgmann, S., Jakobiak, T., Gruber, H., Schroder, H., & Sagel, U., *Ciprofloxacin treatment of urinary infections results in increased resistance of urinary E coli to Ciprofloxacin and Co-trimoxazole.* Polish Journal of Microbiology, 2009. **58**(4): p. 371–373.

28. Bronzwaer, S.L.A.M., Cars, O., Buchholz, U., Molstad, S., Goettsch, W., Veldhuijzen, I.K., Kool, J.L., Sprenger, M.J.W., Degener, J.W. & participants in the European Antimicrobial Resistance Surveillance System, *A European study on the relationship between antimicrobial use and antimicrobial resistance.* Emerging Infectious Diseases, 2002. **8**(3): p. 278–282.

29. Brook, I., *Emergence and persistence of B-lactamase-producing bacteria in the oropharynx following penicillin treatment.* Archives of otolaryngology--head & neck surgery 1988. **114**(6): p. 667–670.

30. Brook, I., & Gober, A.E., *Monthly changes in the rate of recovery of pencillin-resistant organisms from children.* The Pediatric Infectious Disease Journal, 1997. **16**(2): p. 255–257.

31. Brook, I., & Gober, A.E., *Resistance to antimicrobials used for therapy of otitis media and sinusitis: Effect of previous antimicrobial therapy and smoking.* Annals of Otology, Rhinology and Laryngology, 1999. **108**(7): p. 645–647.

32. Butler, C.C., Dunstan, F., Heginbothom, M., Mason, B., Roberts, Z., Hillier, S., Howe, R., Palmer, S., & Howard, A., *Containing antibiotic resistance: decreased antibiotic-resistant coliform urinary tract infections with reduction in antibiotic prescribing by general practices.* British Journal of General Practice, 2007. **57**(543): p. 785–792.

33. Calbo, E., Romani, V., Xercavins, M., Gomez, L., Vidal, C.G., Quintana, S., Vila, J., & Garau, J., *Risk factors for community-onset urinary tract infections due to Escherichia coli harbouring extended-spectrum B-lactamases.* Journal of Antimicrobial Chemotherapy, 2006. **57**(4): p. 780–783.

34. Campbell, K.M., Vaughn, A.F., Russell, K.L., Smith, B., Jimenez, D.L., Barrozo, C.P., Minarcik, J.R., Crum, N.F., Ryan, M.A.K., *Risk factors for community-associated methicillin-resistant Staphyl ococcus aureus infections in an outbreak of disease among military trainees in San Diego, California in 2002.* Journal of Clinical Microbiology, 2004. **42**(9): p. 4050–4053.

35. Chaniotaki, S., Giakouppi, P., Tzouvelekis, L.S., Panagiotakos, D., Kozanitou, M., Petrikkos, G., Avlami, A., the WHONET study group and A.C. Vatopoulos, *Quinolone resistance among Escherichia coli strains from community-acquired urinary tract infections in Greece.* Clinical Microbiology and Infection, 2004. **10**(1): p. 75–78.

36. Charlebois, E.D., Bangsberg, D.R., Moss, N.J., Moore, M.R., Moss, A.R., Chambers, H.F., & Perdreau-Remington, F., *Population-based community prevalence of methicillin-resistant Staphylococcus aureus in the urban poor of San Francisco.* Clinical Infectious Diseases, 2002. **34**(4): p. 425–433.

37. Chiu, S., Ho, P.L., Chow, F.K.H., Yuen, K.L., & Lau, Y.L., *Nasopharyngeal carriage of antimicrobial-resistant Streptococcus pneumoniae among young children attending 79 kindergartens and day care centers in Hong Kong.* Antimicrobial Agents and Chemotherapy, 2001. **45**(10): p. 2765–2770.

38. Chung, A., Perera, R., Brueggemann, A.B., Elamin, A.E., Harnden, A., Mayon-White, R., Smith, S., Crook, D.W., & Mant, D., *Effect of antibiotic prescribing on antibiotic resistance in individual children in primary care: prospective cohort study.* British Medical Journal, 2007. **335**(7617): p. 429–433.

39. Ciftci, E., Dogru, U., Aysev, D., Ince, E., & Guriz, H., *Investigation of risk factors for penicillin-resistant Streptococcus pneumoniae carriage in Turkish children.* Pediatrics International, 2001. **43**(4): p. 385–390.

40. Ciftci, E., Dogru, U., Guriz, H., Aysev, D., & Ince, E., *Investigation of risk factors for tonsillopharyngitis with macrolide resistant Streptococcus pyogenes in Turkish children.* Pediatrics International, 2002. **44**(6): p. 647–651.

41. Cizman, M., Pokorn, M., Seme, K., Orazem, A., & Paragi, M., *The relationship between trends in macrolide use and resistance to macrolides of common respiratory pathogens.* Journal of Antimicrobial Chemotherapy, 2001. **47**(4): p. 475–477.

42. Cizman, M., *The use and resistance to antibiotics in the community.* International Journal of Antimicrobial Agents, 2003. **21**(4): p. 297–307.

43. Clancy, M.J., Graepler, A., Breese, P.E., Price, C.S., & Burman, W.J., *Widespread emergence of methicillin resistance in community-acquired Staphylococcus aureus infections in Denver.* Southern Medical Journal, 2005. **98**(11): p. 1069–1075.

44. Cohen, R., Levy, C., de la Rocque, F., Gelbert, N., Wollner, A., Fritzell, B., Bonnet, E., Tetelboum, R., & Varon, E., *Impact of pneumococcal conjugate vaccine and of reduction of antibiotic use on nasopharyngeal carriage of nonsusceptible pneumococci in children with acute otitis media.* The Pediatric Infectious Disease Journal, 2006. **25**(11): p. 1001–1007.

45. Colgan, R., Johnson, J.J., Kuskowski, M., & Gupta, K., *Risk factors for trimethoprim -sulfamethoxazole resistance in patients with acute uncomplicated cystitis.* Antimicrobial Agents and Chemotherapy, 2008. **52**(3): p. 846–851.

46. Colodner, R., Kometiani, I., Chazan, B., & Raz, R., *Risk factors for community-acquired urinary tract infection due to quinolone-resistance E. Coli.* Infection, 2008. **36**(1): p. 41–45.

47. Como-Sabetti, K., Harriman, K., Fridkin, S., & Lynfield, R., *Community-associated Methicillin-resistant Staphylococcus aureus infection risk factor study.* International Conference on Emerging Infectious Diseases 2008, 2008.

48. Corea, E., de Silva, T., & Perera, J., *Methicillin-resistant Staphylococcus aureus: prevalence, incidence and risk factors associated with colonization in Sri Lanka.* Journal of Hospital Infection, 2003. **55**(2): p. 145–148.

49. Costelloe, C., Metcalfe, C., Lovering, A., Mant, D. & Hay, A.D., *Effect of antibiotic prescribing in primary care on antimicrobial resistance in individual patients: systematic review and meta-analysis.* British Medial Journal, 2010. **340**(7756): c2096.

50. Costelloe, C., Lovering, A., Montgomery, A., Lewis, D., McNulty, C., & Hay, A.D., *Effect of antibiotic prescribing in primary care on methicillin resistant Staphylococcus aureus carriage in community resident adults: a controlled observational study*, in *GRIN*. 2010: Lodz.

51. Craig, A.S., Erwin, P.C., Schaffner, W., Elliot, J.A., Moore, W.L., Ussery, X.T., Patterson, L., Dake, A.D., Hannah, S.G., & Butler, J.C., *Carriage of multidrug-resistant Streptococcus pneumoniae and impact of chemoprophylaxis during an outbreak of meningitis at a day care center.* Clinical Infectious Diseases, 1999. **29**(5): p. 1257–1264.

52. Creech, C.B., Kernodle, D.S., Alsentzer, A., Wilson, C., & Edwards, K.M., *Increasing rates of nasal carriage of methicillin-resistant Staphylococcus aureus in healthy children.* The Pediatric Infectious Disease Journal, 2005. **24**(7): p. 617–621.

53. Dabernat, H., Geslin, P., Megraud, F., Begue, P., Boulesteix, J., Dubreuil, C., de La Roque, F., Trinh, A., & Scheimberg, A., *Effects of cefixime or co-amoxiclav treatment on nasopharyngeal carriage of Streptococcus pneumoniae and Haemophilus influenzae in children with acute otitis media.* Journal of Antimicrobial Chemotherapy, 1998. **41**(2): p. 253–258.

54. Dagan, R., Melamed, R., Muallem, M., Piglansky, L., & Yagupsky, P., *Nasopharyngeal colonization in southern Israel with antibiotic-resistant pneumococci during the first 2 years of life: Relation to serotypes likely to be included in pneumococcal conjugate vaccines.* The Journal of Infectious Diseases, 1996. **174**(6): p. 1352–1355.

55. Dagan, R., Hoberman, A., Johnson, C., Leibovitz, E., Arguedas, A., Rose, F.V., Wynne, B.R., & Jacobs, M.R., *Bacteriological and clinical efficacy of high dose amoxicillin/clavulanate in children with acute otitis media.* The Pediatric Infectious Disease Journal, 2001. **20**(9): p. 829–837.

56. Dagan, R., Barkai, G., Givon-Lavi, N., Sharf, A.Z., Vardy, D., Cohen, T., Lipsitch, M., & Greenberg, D., *Seasonality of antibiotic-resistant Streptococcus pneumoniae that causes acute otitis media: A clue for an antibiotic-restriction policy?* Journal of Infectious Diseases, 2008. **197**(8): p. 1094–1102.

57. Datta, N.F., Reeves, M.C., Brumfitt, W., Orskov, F., & Orskov, I., *R factors in Escherichia coli in faeces after oral chemotherapy in general practice.* The Lancet, 1971. **1**(7694): p. 312–315.

58. Davis, S.L., Perri, M.B., Donabedian, S.M., Manierski, C., Singh, A., Vager, D., Haque, N.Z., Speirs, K., Muder, R.R., Robinson-Dunn, B., Hayden, M.K. & Zervos, M.J., *Epidemiology and outcomes of community-associated methicillin-resistant Staphylococcus aureus infection.* Journal of Clinical Microbiology, 2007. **45**(6): p. 1705–1711.

59. Deeks, S.L., Palacio, R., Ruvinsky, R., Kertesz, D.A., Hortal, M., Rossi, A., Spika, J.S., Di Fabio, J.L., & The Streptococcus pneumoniae working group, *Risk factors and course of illness among children with invasive penicillin-resistant Streptococcus pneumoniae.* Pediatrics, 1999. **103**(2): p. 409–413.

60. Dejthevaporn, C., Vibhagool, A., Thakkinstian, A., Sirinavin, S., & Vorachit, M., *Risk factors for penicillin-resistant Streptococcus pneumoniae acquisition in patients in Bangkok.* Southeast Asian Journal Of Tropical Medicine And Public Health, 2000. **31**(4): p. 679–683.

61. DeLencastre, H., Kristinsson, K. G., Brito-Avo, A., Sanches, I.S., Sa-Leao, R., Saldanha, J., Sigvaldadottir, E., Karlsson, S., Oliviera, D., Mato, R., De Sousa, M.A., & Tomasz, A., *Carriage of respiratory tract pathogens and molecular epidemiology of Streptococcus pneumoniae colonization in healthy children attending day care centers in Lisbon, Portugal.* Microbial Drug Resistance, 1999. **5**(1): p. 19–29.

62. Dellamonica, P., Pradier, C., Leroy, J., Carsenti-Etesse, H., Dupont, M.J., Roussel-Delvallez, M., Dabernat, H., Dunais, B., Martinot, A., Estavoyer, J.M., Grandbastien, B., Guillemot, D., De Bels, F., *Epidemiology and antibiotic susceptibility of nasopharyngeal S pneumoniae and H influenzae isolated from children attending day-care centers in 3 French departments.* Medecine et maladies infectieuses, 2002. **32**(12): p. 650–661.

63. DeNeeling, A.J., Overbeek, B.P., Horrevorts, A.M., Ligtvoet, E.E.J., & Goettsch, W.G., *Antibiotic use and resistance of Streptococcus pneumoniae in The Netherlands during the period 1994–1999.* Journal of Antimicrobial Chemotherapy, 2001. **48**(3): p. 441–444.

64. Dias, R.C., M., *Emergence of invasive erythromycin-resistant Streptococcus pneumoniae strains in Portugal: contribution and phylogenetic relatedness of serotype 14.* Journal of Antimicrobial Chemotherapy, 2004. **54**(6): p. 1035–1039.

65. Dias, R.C., M., *Trends in resistance to penicillin and erythromycin of invasive pneumococci in Portugal.* Epidemiology and Infection, 2008. **136**(7): p. 928–939.

66. Diekama, D.J., Brueggemann, A.B., Doern, G.V., *Antimicrobial drug use and changes in resistance in Streptococcus pneumoniae.* Emerging Infectious Diseases, 2000. **6**(5): p. 552–556.

67. Donnan, P.T., Wei, L., Steinke, D.T., Phillips, G., Clarke, R., Noone, A., Sullivan, F.M., MacDonald, T.M., & Davey, P.G., *Presence of bacteriuria caused by trimethoprim resistant bacteria in patients prescribed antibiotics: multilevel model with practice and individual patient data.* British Medical Journal, 2004. **328**: p. 1297–1301.

68. Doone, J., Klespies, S.L., & Sabella, C., *Risk factors for penicillin-resistant systemic pneumococcal infections in children.* Clinical Pediatrics, 1997. **36**(4): p. 187–191.

69. Dromigny, J.A., Nabeth, P., Juergens-Behr, A., & Perrier-Gros-Claude, J.D., *Risk factors for antibiotic-resistant Escherichia coli isolated from community-acquired urinary tract infections in Dakar, Senegal.* Journal of Antimicrobial Chemotherapy, 2005. **56**(1): p. 236–239.

70. Duchin, J.S., Breiman, R.F., Diamond, A., Lipman, H.B., Block, S.L., Hedrick, J.A., Finger, R., & Elliott, J.A., *High prevalence of multidrug-resistant Streptococcus pneumoniae among children in a rural Kentucky community.* Pediatric Infectious Disease Journal, 1995. **14**(9): p. 745–750.

71. Dueger, E.L., Asturias, E.J., Matheu, J., Gordillo, R., Torres, O., & Halsey, N., *Increasing pencillin and trimethoprim-sulfamethoxazole resistance in nasopharyngeal Streptococcus pneumoniae isolates from Guatemalan children, 2001–2006.* International Journal of Infectious Diseases, 2008. **12**(3): p. 289–297.

72. Duerink, D.O., Lestari, E.S., Hadi, U., Nagelkerke, N.J.D., Severin, J.A., Verbrugh, H. A., Keuter, M., Gyssens, I.C., van den Broek, P.J. on behalf of the study group 'Antimicrobial Resistance in Indonesia: Prevalence and Prevention (AMRIN), *Determinants of carriage of resistant Escherichia coli in the Indonesian population inside and outside hospitals.* Journal of Antimicrobial Chemotherapy, 2007. **60**(2): p. 377–384.

73. Dunais, B., Pradier, C., Carsenti, H., Sabah, M., Mancini, G., Fontas, E., & Dellamonica, P., *Influence of child care on nasopharyngeal carraige of Streptococcus pneumoniae and Haemophilus influenzae.* The Pediatric Infectious Disease Journal, 2003. **22**(7): p. 589–592.

74. ECDC, *Antibiotic Resistance*. 2011. Used Figure 2 to calculate correlations between consumption and resistance. http://www.farnhamdene.com/website/H81615/files/Primary_Care_Factsheet_2011_Antibiotics.pdf

75. European Centre for Disease Prevention and Control. *Annual epidemiological report on communicable diseases in Europe*. 2010. Stockholm: ECDC; 2010.

76. Einarsson, S., Kristjansson, M., Kristinsson, K.G., Kjartansson, G., & Jonsson, S., *Pneumonia caused by penicillin-non-susceptible and penicillin-susceptible pneumococci in adults: A case–control study.* Scandinavian Journal of Infectious Diseases, 1998. **30**(3): p. 253–256.

77. Ellis, M.W., Hospenthal, D.R., Dooley, D.P., Gray, P.J., & Murray, C.K., *Natural history of community-acquired methicillin-resistant Staphylococcus aureus colonization and infection in soldiers.* Clinical Infectious Diseases, 2004. **39**(7): p. 971–979.

78. Ewig, S., Ruiz, M., Torres, A., Marco, F., Martinez, J.A., Sanchez, M., & Mensa, J., *Pneumonia acquired in the community through drug-resistant Streptococcus pneumoniae.* American Journal of Respiratory and Critical Care Medicine, 1999. **159**(6): p. 1835–1842.

79. Fairchok, M.P., Ashton, W.S., & Fischer, G.W., *Carriage of pencillin-resistant pneumococci in a military population in Washington, DC: Risk factors and correlation with clinical isolates.* Clinical Infectious Diseases, 1996. **22**(6): p. 966–972.

80. Finkelstein, J.A., Huang, S.S., Daniel, J., Rifas-Shiman, S.L., Kleinman, K., Goldmann, D., Pelton, S.I., Demaria, A., & Platt, R., *Antibiotic-resistant Streptococcus pneumoniae in the Heptavalent pneumococcal conjugate vaccine era: Predictors of carriage in a multicommunity sample.* Pediatrics, 2003. **112**(4): p. 862–869.

81. Frazee, B.W., Lynn, J., Charlebois, E.D., Lambert, L., Lowery, D., & Perdreau-Remington, F., *High prevalence of methicillin-resistant Staphylococcus aureus in emergency department skin and soft issue infections.* Annals of Emergency Medicine, 2005. **45**(3): p. 311–320.

82. Fritz, S.A., Garbutt, J., Elward, A., Shannon, W., & Storch, G.A., *Prevalence of and risk factors for community-acquired methicillin-resistant and methicillin-sensitive Staphylococcus aureus colonization in children seen in a practice-based research network.* Pediatrics, 2008. **121**(6): p. 1090–1098.

83. Fry, A.M., Jha, H.C., Lietman, T. M., Chaudhary, J.S.P., Bhatta, R.C., Elliott, J., Hyde, T., Schuchat, A., Gaynor, B., Dowell, S.F., *Adverse and beneficial secondary effects of mass treatment with azithromycin to eliminate blindness due to trachoma in Nepal.* Clinical Infectious Diseases, 2002. **35**(4): p. 395–402.

84. Furuno, J.P., McGregor, J.C., Harris, A.D., Johnson, J.A., Johnson, J.K., Langenberg, P., Venezia, R.A., Finkelstein, J., Smith, D.L., Strauss, S.M., & Perencevich, E.N., *Identifying groups at high risk for carriage of antibiotic-resistant bacteria.* Archives of Internal Medicine, 2006. **166**(5): p. 580–585.

85. Gagliotti, C., Nobilio, L., Milandri, M., Moro, M.L., for the Emilia-Romagna Antibiotic Resistance Study Group, *Macrolide prescriptions and erythromycin resistance of Streptococcus pyogenes.* Clinical Infectious Diseases, 2006. **42**(8): p. 1153–1156.

86. Gagliotti, G., Nobilio, L., & Moro, M.L., *Emergence of ciprofloxacin resistance in Escherichia coli isolates from outpatient urine samples.* Clinical Microbiology and Infection, 2007. **13**(3): p. 328–331.

87. Garau, J., Xercavins, M., Rodriguez-Carballeira, M., Gomez-Vera, J.R., Coll, I., Vidal, D., Llovet, T., & Ruiz-Bremon, A., *Emergence and dissemination of quinolone-resistant Escherichia coli in the community.* Antimicrobial Agents and Chemotherapy, 1999. **43**(11): p. 2736–2741.

88. Garcia-Rey, C., Aguilar, L., Bacquero, F., Casal, J., & Martin, J.E., *Pharmacoepidemiological analysis of provincial differences between consumption of macrolides and rates of erythromycin resistance among Streptococcus pyogenes isolates in Spain.* Journal of Clinical Microbiology, 2002. **40**(8): p. 2959–2963.

89. Garcia-Rey, C., Aguilar, L., Baquero, F., Casal, J., & Dal-Re, R., *Importance of local variations in antibiotic consumption and geographical differences of erythromycin and pencillin resistance in Streptococcus pneumoniae.* Journal of Clinical Microbiology, 2002. **40**(1): p. 159–164.

90. Garcia-Rey, C., Fenoll, A., Aguilar, L., & Casal, J., *Effect of social and climatological factors on antimicrobial use and Streptococcus pneumoniae resistance in different provinces in Spain.* Journal of Antimicrobial Chemotherapy, 2004. **54**(2): p. 465–471.

91. Gaynor, B.D., Chidambaram, J.D., Cevallos, V., Miao, Y., Miller, K., Jha, H. C., Bhatta, R.C., Chaudhary, J.S.P., Holm, S.O., Whitcher, J.P., Holbrook, K.A., Fry, A.M., & Lietman, T.M. , *Topical ocular antibiotics induce bacterial resistance at extraocular sites.* British Journal of Ophthalmology, 2005. **89**(9): p. 1097–1099.

92. Gehanno, P., N'guyen, L., Derriennic, M., Pichon, F., Goehrs, J., & Berche, P., *Pathogens isolated during treatment failures in otitis.* The Pediatric Infectious Disease Journal, 1998. **17**(10): p. 885–890.

93. Ghaffar, F., Muniz, L.S., Katz, K., Reynolds, J., Smith, J.L., Davis, P., Friedland, I.R., & McCracken, G.H., *Effects of amoxicillin/clavulanate or azithromycin on nasopharyngeal carriage of Streptococcus pneumoniae and Haemophilus influenzae in children with acute otitis media.* Clinical Infectious Diseases, 2000. **31**(4): p. 875–880.

94. Gobernardo, M., Valdes, L., Alos, J.I., Garcia-Rey, C., Dal-Re, R., Garcia-de-Lomas, J., and the Spanish Surveillance Group for Urinary Pathogens, *Antimicrobial susceptibility of clinical Escherichia coli isolates from uncomplicated cystitis in women over a 1-year period in Spain.* Rev Esp Quimioterap, 2007. **20**(1): p. 68–76.

95. Goettsch, W., van Pelt, W., Nagelkerke, N., Hendrix, M.G.R., Buiting, A.G.M., Petit, P.L., Sabbe, L.J.M., van Griethuysen, A.J.A., & de Neeling, A.J., *Increasing resistance to fluoroquinolones in Escherichia coli from urinary tract infections in The Netherlands.* Journal of Antimicrobial Chemotherapy, 2000. **46**(2): p. 223–228.

96. Gomez-Barreto, D., Calderon-Jaimes, E., Rodriguez, R.S., Espinosa, L.E., Vina-Flores, L., & Jimenez-Rojas, V., *Carriage of antibiotic-resistant pneumococci in a cohort of a daycare center.* Salud Publica de Mexico, 2002. **44**(1): p. 26–32.

97. Goossens, H., Lammens, C., Lontie, M., Stalpaert, M., Andre, M., Van Pelt, H., Dellanoy, P., Drion, S., & Hendrickx, E., *Streptococcus pyogenes (S Py) resistance to erythromycin (ERY) in relation to mechanism of resistance, genotype, and comsumption of macrolides (M)* Abstracts of the 41st Interscience Conference on Antimicrobial Agents and Chemotherapy, 2001: p. 134–135 Abstract C2-1314.

98. Goossens, H., Ferech, M., Vander Stichele, R. & Elseviers, M., *Outpatient antibiotic use in Europe and association with resistance: a cross-national database study.* The Lancet, 2005. **365**(9459): p. 579–587.

99. Gottesman, B.S., Carmeli, Y., Shitrit, P., & Chowers, M., *Impact of quinolone restriction on resistance patterns of Escherichia coli isolated from urine by culture in a community setting.* Clinical Infectious Diseases, 2009. **49**(6): p. 869–875.

100. Granizo, J.J., Aguilar, L., Casal, J., Garcia-Rey, C., Dal-Re, R. & Baquero, F., *Streptococcus pneumoniae resistance to erythromycin and penicillin in relation to macrolide and B-lactam consumption in Spain (1979–1997)* Journal of Antimicrobial Chemotherapy, 2000. **46**(5): p. 767–773.

101. Granizo, J.J., Aguilar, L., Casal, J., Dal-Re, R. & Baquero, F., *Streptococcus pyogenes resistance to erythromycin in relation to macrolide consumption in Spain (1986–1997).* Journal of Antimicrobial Chemotherapy, 2000. **46**(6): p. 959–964.

102. Greenberg, D., Givon-Lavi, N., Sharf, A. Z., Vardy, D., & Dagan, R., *The association between antibiotic use in the community and nasopharyngeal carriage of antibiotic-resistant Streptococcus pneumoniae in Bedouin children.* The Pediatric Infectious Disease Journal, 2008. **27**(9): p. 776–782.

103. Grivea, I.N., Tsantouli, A. G., Chryssanthopoulou, D.C., & Syrogiannopoulos, G.A., *Interaction of the heptavalent pneumococcal conjugate vaccine and the use of individial antiibotics among children on nasopharyngeal colonization with erythromycin-resistant Streptococcus pneumoniae.* European Journal of Clinical Microbiology and Infectious Diseases, 2010. **29**(1): p. 97–105.

104. Groom, A.V., Wolsey, D.H., Naimi, T.S., Smith, K., Johnson, S., Boxrud, D., Moore, K.A., & Cheek, J.A., *Community-acquired methicillin-resistant Staphylococcus aureus in a rural American Indian community.* Journal of the American Medical Association, 2001. **286**(10): p. 1201–1205.

105. Guillemot, D., Carbon, C., Balkau, B., Geslin, P., LeCoeur, H., Vauzelle-Kervroedan, F., Bouvenot, G., & Eschwege, E., *Low dosage and long treatment duration of B-lactam Risk factors for carriage of pencillin-resistant Streptococcus pneumoniae.* Journal of the American Medical Association, 1998. **279**(5): p. 365–370.

106. Guillemot, D., Boutou, O., Carbon, C., Balkau, B., Geslin, P., Lecoeur, H., Weber, P., & Eschwege, E., *Pencillin-resistant Streptococcus pneumoniae (PRSp) carriage is strongly related to B-lactam use*, in *Thirty-Eighth Interscience Conference on Antimicrobial Agents and Chemotherapy* 1998, American Society for Microbiology: San Diego, CA USA. p. 75.

107. Guillemot, D., Varon, E., Bernede, C., Weber, P., Henriet, L., Simon, S., Laurent, C., Lecoeur, H., & Carbon, C., *Reduction in antibiotic use in the community reduces the rate of colonization with pencillin G-nonsusceptible Streptococcus pneumoniae.* Clinical Infectious Diseases, 2005. **41**(7): p. 930–938.

108. Gunnarsson, O., & Ekdahl, K., *Previous respiratory tract infections and antibiotic consumption in children with long- and short-term carriage of penicillin-resistant Streptococcus pneumoniae.* Epidemiology and Infection, 1998. **121**(3): p. 523–528.

109. Gupta, K., Hooton, T.M., & Stamm, W.E., *Isolation of fluoroquinolone-resistant rectal Escherichia coli after treatment of acute uncomplicated cystitis.* Journal of Antimicrobial Chemotherapy, 2005. **56**(1): p. 243–246.

110. Hannah, E.L., Angulo, F.J., Johnson, J.R., Haddadin, B., Williamson, J., & Samore, M.H. , *Drug-resistant Escherichia coli, rural Idaho.* Emerging Infectious Diseases, 2005. **11**(10): p. 1614–1617.

111. Harbarth, S., Sax, H., Fankhauser-Rodriguez, C., Schrenzel, J., Agostinho, A., & Pittet, D., *Evaluating the probability of previously unknown carriage of MRSA at hospital admission.* The American Journal of Medicine, 2006. **119**(3): p. e15-e23.

112. Hay, A.D., Thomas, M., Montgomery, A., Wetherell, M., Lovering, A., McNulty, C., Lewis, D., Carron, B., Henderson, E., & MacGowan, A., *The relationship between primary care antibiotic prescribing and bacterial resistance in adults in the community: a controlled observational study using individual patient data.* Journal of Antimicrobial Chemotherapy, 2005. **56**(1): p. 146–153.

113. Hennessy, T.W., Petersen, K.M., Bruden, D., Parkinson, A.J., Hurlburt, D., Getty, M., Schwartz, B., & Butler, J.C., *Changes in antibiotic-prescribing practices and carriage of penicillin-resistant Streptococcus pneumoniae: A controlled intervention trial in rural Alaska.* Clinical Infectious Diseases, 2002. **34**(12): p. 1543–1550.

114. Hennessy, T.W., Bruden, D., Petersen, K.M., Parkinson, A.J., Hurlburt, D., Getty, M., Butler, J.C., & Schwartz, B, *Effect of high-dose amoxicillin on the prevalence of penicillin-resistant Streptococcus pneumoniae in rural Alaska.* The Journal of the American Medical Association, 2002. **287**(16): p. 2078–2079.

115. Hidron, A.I., Kourbatova, E.V., Halvosa, J.S., Terrell, B.J., McDougal, L.K., Tenover, F.C., Blumberg, H.M., & King, M.D., *Risk factors for colonization with methicillin-resistant Staphylococcus aureus (MRSA) in patients admitted to an urban hospital: Emergence of community-associated MRSA nasal carriage.* Clinical Infectious Diseases, 2005. **41**(2): p. 159–166.

116. Hillier, S., Roberts, Z., Dunstan, F., Butler, C., Howard, A., & Palmer, S., *Prior antibiotics and risk of antibiotic-resistant community-acquired urinary tract infection: a case–control study.* Journal of Antimicrobial Chemotherapy, 2007. **60**(1): p. 92–99.

117. Hjaltested, E.K.R., Bernatoniene, J., Erlendsdottir, H., Kaltenis, P., Bernatoniene, G., Gudnason, T., Haraldsson, A., & Kristinsson, K.G., *Resistance in respiratory tract pathogens and antimicrobial use in Icelandic and Lithuanian children.* Scandinavian Journal of Infectious Diseases, 2003. **35**(1): p. 21–26.

118. Ho, P., Yip, K., Chow, K., Lo, J.Y.C., Que, T., & Yuen, K., *Antimicrobial resistance among uropathogens that cause acute uncomplicated cystitis in women in Hong Kong: a prospective multicenter study in 2006 to 2008.* Diagnostic microbiology and infectious disease, 2010. **66**(1): p. 87–93.

119. Hoberman, A., Paradise, J.L., Block, S., Burch, D.J., Jacobs, M.R., Balanescu, M.I., *Efficacy of amoxicillin/clavulanate for acute otitis media: Relation to Streptococcus pneumoniae susceptiblity.* The Pediatric Infectious Disease Journal, 1996. **15**(10): p. 955–962.

120. Howard, A.J., Magee, J.T., Fitzgerald, K.A., & Dunstan, F.D.J., *Factors associated with antibiotic resistance in coliform organisms from community urinary tract infections in Wales.* Journal of Antimicrobial Chemotherapy, 2001. **47**(3): p. 305–313.

121. Hsueh, P., Shyr, J., & Wu, J., *Decreased erythromycin use after antimicrobial reimbursement restriction for undocumented bacterial upper respiratory tract infections significantly reduced erythromycin resistance in Streptococcus pyogenes in Taiwan.* Clinical Infectious Diseases, 2005. **40**(6): p. 903–905.

122. Hsueh, P.R., *Decreasing rates of resistance to pencillin, but not erythromycin, in Streptococcus pneumoniae after introduction of a policy to restrict antibiotic use in Taiwan.* Clinical Microbiology and Infection, 2005. **11**(11): p. 925–927.

123. Hsueh, P.R., Shyr, J.M., & Wu, J.J., *Changes in macrolide resistance among respiratory pathogens after decreased erythromycin consumption in Taiwan.* Clinical Microbiology and Infection, 2006. **12**(3): p. 296–298.

124. Huang, W.H., P., *Methicillin-resistant Staphylococcus aureus infections in acute rhinosinusitis.* The Laryngoscope, 2006. **116**(2): p. 288–291.

125. Hyatt, J.M., Bhavnani, S.M., Forrest, A., Faden, H., & Schentag, J.J., *Factors associated with changes in nasopharyngeal strains of Streptococcus pneumoniae in children from birth through two years of age*, in *Thirty-Eight Interscience Conference on Antimicrobial Agents and Chemotherapy*. 1998, American Society for Microbiology: San Diego, CA USA. p. 74.

126. Jackson, M.A., Shelton, S., Nelson, J.D., & McCracken, G.H., *Relatively penicillin-resistant pneumococcal infections in pediatric patients.* Pediatric Infectious Disease, 1984. **3**(2): p. 129–132.

127. Jakobsson, H., Wreiber, K., Fall, K., Fjelstad, B., Nyren, O., & Engstrand, L., *Macrolide resistance in the normal microbiota after Helicobacter pylori treatment.* Scandinavian Journal of Infectious Diseases, 2007. **39**(9): p. 757–763.

128. Jensen, U.S., Muller, A., Brandt, C.T., Frimodt-Moller, N., Hammerum, A.M., & Monnet, D. L. on behalf of the DANRES study group, *Effect of generics on price and consumption of ciprofloxacin in primary healthcare: the relationship to increasing resistance.* Journal of Antimicrobial Chemotherapy, 2010. **65**(6): p. 1286–1291.

129. Jernigan, J.A., Pullen, A.L., Partin, C., & Jarvis, W.R., *Prevalence of and risk factors for colonization with methicillin-resistant Staphylococcus aureus in an outpatient clinic population.* Infection Control and Hospital Epidemiology, 2003. **24**(6): p. 445–450.

130. Jindrak, V., Marek, J., Vanis, V., Urbaskova, P., Vlcek, J., Janiga, L., & Maresova, V., *Improvements in antibiotic prescribing by community paediatricians in the Czech Republic.* Euro Surveillance, 2008. **13**(46): p. pii = 19040.

131. Johnson, S.R., Thompson, R.C.F., Humphreys, H., & Macfarlane, J.T., *Clinical features of patients with B-lactamase producing Haemophilus influenzae isolated from sputum.* Journal of Antimicrobial Chemotherapy, 1996. **38**(5): p. 881–884.

132. Kahlmeter, G., Menday, P., & Cars, O., *Non-hospital antimicrobial use and resistance in community-acquired Escherichia coli urinary tract infection.* Journal of Antimicrobial Chemotherapy, 2003. **52**(6): p. 1005–1010.

133. Kahlmeter, G., *Prevalence and antimicrobial susceptbility of pathogens in uncomplicated cystitis in Europe. The ECO-SENS study.* International Journal of Antimicrobial Agents, 2003. **22**(Supplement 2): p. S49-S52.

134. Kalter, H.D., Gilman, R.H., Moulton, L.H., Cullotta, A.R., Cabrera, L., & Velapatino, B., *Risk factors for antibiotic-resistant Escherichia coli in young children in Peru: community-based cross-sectional prevalence study.* The American Journal of Tropical Medicine and Hygiene, 2010. **82**(5): p. 879–888.

135. Kandemir, O., Akbay, E., Sahin, E., Milcan, A., & Gen, R., *Risk factors for infection of the diabetic foot with multi-antibiotic resistant microorganisms.* Journal of Infection, 2007. **54**(5): p. 439–445.

136. Karpanoja, P., Nyberg, S.T., Bergman, M., Voipio, T., Paakkari, P., Huovinen, P., Sarkkinen, H., & the Finnish Study Group for Antimicrobial Resistance (FiRe Network), *Connection between trimethoprim-sulfamethoxazole use and resistance in Streptococcus pneumoniae Haemophilus influenzae, and Moraxella catarrhalis.* Antimicrobial Agents and Chemotherapy, 2008. **52**(7): p. 2480–2485.

137. Katsarolis, I., Poulakou, G., Analitis, A., Matthaiopoulou, I., Roilides, E., Antachopoulos, C., Kafetzis, D.A., Daikos, G.I., Vorou, R., Koubaniou, C., Pneumatikos, I., Samonis, G., Syriopoulou, V., Giamarellou, H., & Kanellakopoulou, K., *Risk factors for nasopharyngeal carriage of drug-resistant Streptococcus pneumoniae: data from a nation-wide surveillance study in Greece.* BMC Infectious Diseases, 2009. **9**(1): p. 120–129 ?

138. Kellner, J.D., Ford-Jones, E.L., & Members of the Toronto Child Care Centre Study Group, *Streptococcus pneumoniae carriage in children attending 59 Canadian child care centers.* Archives of Pediatrics and Adolescent Medicine, 1999. **153**(5): p. 495–502.

139. Kristiansson, C., Grape, M., Gotuzzo, E., Samalvides, F., Chauca, J., Larsson, M., Bartoloni, A., Pallecchi, L., Kronvall, G., & Petzold, M., *Socioeconomic factors and antibiotic use in relation to antimicrobial resistance in the Amazonian area of Peru.* Scandinavian Journal of Infectious Diseases, 2009. **41**(4): p. 303–312.

140. Kronenberger, C.B., Hoffman, R.E., Lezotte, D.C., & Marine, W.M., *Invasive penicillin-resistant pneumococcal infections: A prevalence and historical cohort study.* Emerging Infectious Diseases, 1996. **2**(2): p. 121–124.

141. Kwan, G., T.S., Davis, R.L., Shay, D.K., Black, S., Shinefield, H., & Koepsell, T., *Is household antibiotic use a risk factor for antibiotic-resistant pneumococcal infection?* Epidemiology and Infection, 2002. **129**(3): p. 499–506.

142. Leach, A.J., Shelby-James, T.M., Mayo, M., Gratten, M., Laming, A.C., Currie, B.J., & Mathews, J.D., *A prospective study of the impact of community-based azithromycin treatment of trachoma on carriage and resistance of Streptococcus pneumoniae.* Clinical Infectious Diseases, 1997. **24**(3): p. 356–362.

143. Lee, D.S., Lee. C.B., & Lee. S., *Prevalence and risk factors for extended spectrum beta-lactamase-producing uropathogens in patients with urinary tract infection.* Korean Journal of Urology, 2010. **51**(7): p. 492–497.

144. Leflon-Guibout, V., Ternat, G., Heym, B., & Nicolas-Chanoine, M., *Exposure to co-amoxiclav as a risk factor for co-amoxiclav-resistant Escherichia coli urinary tract infection.* Journal of Antimicrobial Chemotherapy, 2002. **49**(2): p. 367–371.

145. Leman, R., Alvarado-Ramy, F., Pocock, S., Barg, N., Kellum, M., McAllister, S., Cheek, J., & Kuehnert, M., *Nasal carriage of methicillin-resistant Staphylococcus aureus in an American Indian population.* Infection Control and Hospital Epidemiology, 2004. **25**(2): p. 121–125.

146. Lescure, F., Locher, G., Eveillard, M., Biendo, M., Van Agt, S., Le Loup, G., Douadi, Y., Ganry, O., Vandenesch, F., Eb, F., Schmit, J., & Etienne, J., *Community-acquired infection with healthcare-associated methicillin-resistant Staphylococcus aureus: The role of home nursing care.* Infection Control and Hospital Epidemiology, 2006. **27**(11): p. 1213–1218.

147. Lietzau, S., Sturmer, T., Erb, A., von Baum, H., Marre, R., & Brenner, H., *Prevalence and determinants of nasal colonization with antibiotic-resistant Staphylococcus aureus among unselected patients attending general practitioners in Germany.* Epidemiology and Infection, 2004. **132**(4): p. 655–662.

148. Lietzau, S., Hoewner, M., von Baum, H., Marre, R., & Brenner, H., *Antibiotic resistant fecal isolates of Enterococci among unselected patients outside the clinical sector: an epidemiological study from Southern Germany.* Pharmacoepidemiology and Drug Safety, 2006. **15**(4): p. 275–277.

149. Lietzau, S., Raum, E., von Baum, H., Marre, R., & Brenner, H., *Clustering of antibiotic resistance of E coli in couples: suggestion for a major role of conjugal transmission.* BMC Infectious Diseases, 2006. **6**(1): p. 119–123.

150. Lietzau, S., Raum, E., von Baum, H., Marre, R., & Brenner, H., *Household contacts were key factor for children's colonization with resistant Escherichia coli in community setting.* Journal of Clinical Epidemiology, 2007. **60**(11): p. 1149–1155.

151. Livermore, D.M., Stephens, P., Weinberg, J., Johnson, A.P., Gifford, T., Northcutt, D., James, D., George, R.C. & Speller, D.C.E., *Regional variation in ampicillin and trimethoprim resistance in Escherichia coli in England from 1990 to 1997, in relation to bacterial prescribing.* Journal of Antimicrobial Chemotherapy, 2000. **46**(3): p. 411–422.

152. Livermore, D.M., Reynolds, R., Stephens, P., Duckworth, G., Felmingham, D., Johnson, A.P., Murchan, S., Murphy, O., Gungabissoon, U., Waight, P., Pebody, R., Shackcloth, J., Warner, M., Williams, L., & George, R.C., *Trends in penicillin and macrolide resistance among pneumococci in the UK and the Republic of Ireland in relation to antibiotic sales to pharmacies and dispensing doctors.* International Journal of Antimicrobial Agents, 2006. **28**(4): p. 273–279.

153. Lucet, J., Chevret, S., Durand-Zaleski, I., Chastang, C., & Regnier, B., *Prevalence and risk factors for carriage of methicillin-resistant Staphlococcus aureus at admission to the intensive care unit.* Archives of Internal Medicine, 2003. **163**(2): p. 181–188.

154. Magee, J.T., Pritchard, E.L., Fitzgerald, K.A., Dunstan, D.J. & Howard, A.J., *Antibiotic prescribing and antibiotic resistance in community practice: retrospective study, 1996–8.* British Medial Journal, 1999. **319**(7219): p. 1239–1240.

155. Malhotra-Kumar, S., Lammens, C., Coenen, C., Van Herck, K., & Goossens, H, *Effect of azithromycin and clarithromycin therapy on pharyngeal carriage of macrolide-resistant streptococci in healthy volunteers: a randomised, double-blind, placebo-controlled study.* The Lancet, 2007. **369**(9560): p. 482–490.

156. Marton, A.M., Z., *Epidemiological studies on drug resistance in Streptococcus pneumoniae in Hungary: An update for the 1990s.* Microbial Drug Resistance, 1999. **5**(3): p. 201–205.

157. McMahon, B.J., Hennessey, T.W., Bensler, M., Bruden, D.L., Parkinson, A.J., Morris, J.M., Reasonover, A.L., Hurlburt, D.A., Bruce, M.G., Sacco, F., & Butler, J.C., *The relationship between previous antimicrobial use, antimicrobial resistance, and treatment outcomes for Helicobacter pylori infections.* Annals of Internal Medicine, 2003. **139**(6): p. 463–469.

158. Melander, E., Molstad, S., Persson, K., Hansson, H.B., Soderstrom, M., & Ekdahl, K., *Previous antibiotic consumption and other risk factors for carriage of penicillin-resistant Streptococcus pneumoniae in children.* European Journal of Clinical Microbiology and Infectious Diseases, 1998. **17**(12): p. 834–838.

159. Melander, E., Ekdahl, K., Jonsson, G., & Molstad, S., *Frequency of penicillin-resistant pneumococci in children is correlated to community utilization of antibiotics.* Pediatric Infectious Disease Journal, 2000. **19**(12): p. 1172–1177.

160. Mera, R.M., Miller, L.A., & White, A., *Antibacterial use and Streptococcus pneumoniae pencillin resistance: A temporal relationship model.* Microbial Drug Resistance, 2006. **12**(3): p. 158–163.

161. Metlay, J.P., Strom, B.L. & Asch, D.A., *Prior antimicrobial drug exposure: a risk factor for trimethoprim-sulfamethoxazole-resistant urinary tract infections.* Journal of Antimicrobial Chemotherapy, 2003. **51**(4): p. 963–970.

162. Miller, L.G., Perdreau-Remington, F., Bayer, A.S., Diep, B., Tan, N., Bharadwa, K., Tsui, J., Perlroth, J., Shay, A., Tagudar, G., Ibebuogu, U., & Spellberg, B., *Clinical and epidemiologic characteristics cannot distinguish community-associated methicillin-resistant Staphylococcus aureus infection from methicillin-susceptible S aureus infection: A prospective investigation.* Clinical Infectious Diseases, 2007. **44**(4): p. 471–482.

163. Molstad, S., Eliasson, I., Hovelius, B., Kamme, C., & Schalen, C., *Beta-lactamase production in the upper respiratory tract flora in relation to antibiotic consumption: A study in children attending day nurseries.* Scandinavian Journal of Infectious Diseases, 1988. **20**(3): p. 329–334.

164. Moore, M.R., Hyde, T.B., Hennessy, T.W., Parks, D.J., Reasonver, A.L., Harker-Jones, M., Gove, J., Bruden, D.L., Rudolph, K., Parkinson, A., Butler, J.C., & Schuchat, A., *Impact of a conjugate vaccine on community-wide carriage of nonsusceptible Streptococcus pneumoniae in Alaska.* The Journal of Infectious Diseases, 2004. **190**(11): p. 2031–2038.

165. Moran, G.J., Krishnadasan, A., Gorwitz, R.J., Fosheim, G.E., McDougal, L.K., Carey, R.B., & Talan, D.A., *Methicillin-resistant S aureus infections among patients in the emergency department.* The New England Journal of Medicine, 2006. **355**(7): p. 666–674.

166. Moreno, F., Crisp, C., Jorgensen, J.H., Patterosn, J.E., *Methicillin-resistant Staphylococcus aureus as a community organism.* Clinical Infectious Diseases, 1995. **21**(5): p. 1308–1312.

167. Moreno, F., Garcia-Leoni, M.E., Cercenado, E., Diaz, M.D., Bernaldo de Quiros, J.C.L., & Bouza, E., *Infections caused by erythromycin-resistant Streptococcus pneumoniae: Incidence, risk factors, and response to therapy in a prospective study.* Clinical Infectious Diseases, 1995. **20**(5): p. 1195–1200.

168. Nasrin, D., Collignon, P.J., Roberts, L., Wilson, E.J., Pilotto, L.S., Douglas, R.M., *Effect of B lactam antibiotic use in children on pneumococcal resistance to penicillin: prospective cohort study.* British Medical Journal, 2002. **324**(7328): p. 28–30.

169. Nava, J.M., Bella, F., Garau, J., Lite, J., Morera, M., Marti, C., Fontanals, D., Font, B., Pineda, V., Uriz, S., Deulofeu, F., Calderon, A., Duran, P., Grau, M., & Agudo, A., *Predictive factors for invasive disease due to pencillin-resistant Streptococcus pneumoniae: A population-based study.* Clinical Infectious Diseases, 1994. **19**(5): p. 884–890.

170. Neto, A.S., Lavado, P., Flores, P., Dias, R., Pessanha, M.A., Sousa, E., Palminha, J.M., Canica, M., & Esperanca-Pina, J. , *Risk factors for the nasopharyngeal carriage of respiratory pathogens by Portuguese children: Phenotype and antimicrobial susceptibility of Haemophilus influenzae and Streptococcus pneumoniae.* Microbial Drug Resistance, 2003. **9**(1): p. 99–108.

171. Neuman, M.I., Kelley, M., Harper, M.B., File, T.M., & Camargo, C.A., *Factors associated with antimicrobial resistance and mortality in pneumococcal bacteremia.* The Journal of Emergency Medicine, 2007. **32**(4): p. 349–357.

172. Nicoletti, J., Kuster, S.P., Sulser, T., Zbinden, R., Ruef, C., Ledergerber, B., & Weber, R., *Risk factors for urinary tract infections due to ciprofloxacin-resistant Escherichia coli in a tertiary care urology department in Switzerland.* Swiss Medical Weekly, 2010. **140**: p. E1-E8.

173. Nielsen, H.U.K., Monet, D.L., Sorensen, T.L., Kaltoft, M., Konradsen, H.B., & Frimodt-Moller, N., *Relationship between antimicrobial use and rate of invasive erythromycin-resistant Streptococcus pneumoniae at county level.* Abstracts of the 41st Interscience Conference on Antimicrobial Agents and Chemotherapy, 2001: p. page 137 Abstract C2-1867.

174. Nielsen, H.U.K.H., A.M., Ekelund, K., Bang, D., Pallesen, L.V., & Frimodt-Moller, N., *Tetracycline and macrolide co-resistance in Streptococcus pyogenes: Co-selection as a reason for increase in macrolide-resistant S pyogenes?* Microbial Drug Resistance, 2004. **10**(3): p. 231–238.

175. Nilsson, P.L., M.H., *Impact of socioeconomic factors and antibiotic prescribing on pencillin-non-susceptible Streptococcus pneumoniae in the city of Malmo.* Scandinavian Journal of Infectious Diseases, 2005. **37**(6–7): p. 436–441.

176. Nissinen, A., Gronroos, P., Huovinen, P., Herva, E., Katila, M., Klaukka, T., Kontiainen, S., Liimatainen, O., Oinonen, S., & Makela, P., *Development of B-lactamase mediated resistance to pencillin in middle-ear isolates of Moraxella catarrhalis in Finnish children, 1978–1993.* Clinical Infectious Diseases, 1995. **21**(5): p. 1193–1196.

177. NORM/NORM-VET, *Usage of antimicrobial agents and occurrence of antimicrobial resistance in Norway*. 2009. http://www.vetinst.no/eng/Publications/Norm-Norm-Vet-Report/Norm-Norm-Vet-report-2009

178. Ortega, M., Marco, F., Soriano, A., Almela, M., Martinez, J.A., Munoz, A., & Mensa, J., *Analysis of 4758 Escherichia coli bacteraemic episodes: predictive factors for isolation of an antibiotic-resistant strain and their impact on the outcome.* Journal of Antimicrobial Chemotherapy, 2009. **63**(3): p. 568–574.

179. Panhotra, B.R., Saxena, A.K., & Al Mulhim, A.S., *Prevalence of methicillin-resistant and methicillin-sensitive Staphylococcus aureus nasal colonization among patients at the time of admission to the hospital.* Annals of Saudi Medicine, 2005. **25**(4): p. 304–308.

180. Pedersen, G., Schonheyder, H.C., Steffensen, F.H., & Sorensen, H.T., *Risk of resistance related to antibiotic use before admission in patients with community-acquired bacteraemia.* Journal of Antimicrobial Chemotherapy, 1999. **43**(1): p. 119–126.

181. Pedersen, G., Schonheyder, H.C., Kristensen, B., & Sorensen, H.T., *Community-acquired bacteraemia and antibiotic resistance.* Danish Medical Bulletin, 2000. **47**(4): p. 296–300.

182. Pena, C., Albareda, J.M., Pallares, R., Pujol, M., Tubau, F., & Ariza, J., *Relationship between quinolone use and emergence of ciprofloxacin-resistant Escherichia coli in bloodstream infections.* Antimicrobial Agents and Chemotherapy, 1995. **39**(2): p. 520–524.

183. Petrosillo, N., Pantosti, A., Bordi, E., Spano, A., Del Grosso, M., Tallarida, B., & Ippolito, G., *Prevalence, determinants, and molecular epidemiology of Streptococcus pneumoniae isolates colonizing the nasopharynx of healthy children in Rome.* European Journal of Clinical Microbiology and Infectious Diseases, 2002. **21**(3): p. 181–188.

184. Pihlajamaki, M., Kotilainen, P., Kaurila, T., Klaukka, T., Palva, E., Huovinen, P. & the Finnish Study Group for Antimicrobial Resistance (FiRe Network), *Macrolide-resistant Streptococcus pneumoniae and use of antimicrobial agents.* Clinical Infectious Diseases, 2001. **33**(4): p. 483–488.

185. Pradier, C., Dunais, B., Largillier, R., Carsenti-Etesse, H., Bernard, E., Scheimberg, A., & Dellamonica, P., *Nasopharyngeal carriage of Streptococcus pneumoniae in children's day-care centers: 10-month follow-up study in Nice, France.* Clinical Microbiology and Infection, 1997. **3**(6): p. 705–708.

186. Priest, P., Yudkin, P., McNulty. C. & Mant, D., *Antibacterial prescribing and antibacterial resistance in English general practice: cross sectional study.* British Medical Journal, 2001. **323**(7320): p. 1037–1041.

187. Radetsky, M.S., Istre, G.R., Johansen, T.L., Parmelee, S.W., Lauer, B.A., Wiesenthal, A.M., & Glode, M.P., *Multiply resistant pneumococcus causing meningitis: its epidemiology within a day-care center.* The Lancet, 1981. **2**(8250): p. 771–773.

188. Raz, R., Hefter, H. Oren, B. Kennes, Y., *Antimicrobial resistance of urinary isolates in the community and its relation to antibiotic use.* Israel Journal of Medical Sciences, 1993. **29**(4): p. 207–210.

189. Raz, R., Okev, N., Kennes, Y., Gilboa, A., Lavi, I., & Bisharat, N., *Demographic characteristics of patients with community-acquired bacteriuria and susceptibility of urinary pathogens to antimicrobials in Northern Israel* IMAJ, 2000. **2**(6): p. 426–429.

190. Ready, D., Lancaster, H., Qureshi, F., Bedi, R., Mullany, P., & Wilson, M., *Effect of amoxicillin use on oral microbiota in young children.* Antimicrobial Agents and Chemotherapy, 2004. **48**(8): p. 2883–2887.

191. Regev-Yochay, G., Raz, M., Shainberg, B., Dagan, R., Varon, M., Dushenat, M., & Rubinstein, E., *Independent risk factors for carriage of pencillin-non-susceptible Streptococcus pneumoniae.* Scandinavian Journal of Infectious Diseases, 2003. **35**(4): p. 219–222.

192. Reichler, M.R., Allphin, A.A., Breiman, R.F., Schreiber, J.R., Arnold, J.E., McDougal, L.K., Facklam, R.R., Boxerbaum, B., May, D., Walton, R. O., & Jacobs, M.R., *The spread of multiply resistant Streptococcus pneumoniae at a day care center in Ohio.* The Journal of Infectious Diseases, 1992. **166**(6): p. 1346–1353.

193. Reichler, M.R., Rakovsky, J., Sobotova, A., Slacikova, M., Hlavacova, B., Hill, B., Krajcikova, L., Tarina, P., Facklam, R.R., & Breiman, R.F., *Multiple antimicrobial resistance of pneumococci in children with otitis media, bacteremia, and meningitis in Slovakia.* International Journal of Pediatric Otorhinolaryngology, 1996. **34**(3): p. 288–289.

194. Reinert, R.R., Al-Lahman < A., Lemperie, M., Tenholte, C., Briefs, C., Haupts, S., Hubert Gerards, H., & Lutticken, R., *Emergence of macrolide and penicillin resistance among invasive pneumococci isolates in Germany.* Journal of Antimicrobial Chemotherapy, 2002. **49**(2): p. 61–68.

195. Renneberg, J.R., V.T., *Epidemiological studies of pencillin resistance in Danish Staphylococcus aureus strains in the period 1977–1990.* Scandinavian Journal of Infectious Diseases, 1992. **24**(4): p. 401–409.

196. Rezende, N.A., Blumberg, H.M., Metzger, B.S., Larsen, N.M., Ray, S.M., & McGowan, J.E., *Risk factors for methicillin-resistance among patients with Staphylococcus aureus bacteremia at the time of hospital admission.* The American Journal of the Medical Sciences, 2002. **323**(3): p. 117–123.

197. Ridgway, E.J., Tremlett, C.H., & Allen, K.D., *Capsular serotypes and antibiotic sensitivity of Streptococcus pneumoniae isolated from primary-school children.* Journal of Infection, 1995. **30**(3): p. 245–251.

198. Riedel, S., Beekman, S.E., Heilmann, K.P., Richter, S.S., Garcia-de-Lomas, J., Ferech, M., Goosens, H., & Doern, G.V., *Antimicrobial use in Europe and antimicrobial resistance in Streptococcus pneumoniae.* European Journal of Clinical Microbiology and Infectious Diseases, 2007. **26**(7): p. 485–490.

199. Ruhe, J.J., & Hasbun, R., *Streptococcus pneumoniae bacteremia: Duration of previous antibiotic use and association with penicillin resistance.* Clinical Infectious Diseases, 2003. **36**(9): p. 1132–1138.

200. Ruhe, J.J., Myers, L., Mushatt, D., & Hasbun, R., *High-level penicillin-nonsusceptible Streptococcus pneumoniae bacteremia: Identification of a low-risk subgroup.* Clinical Infectious Diseases, 2004. **38**(4): p. 508–514.

201. Saah, A.J., Mallonee, J.P., Tarpay, M., Thornsberry, C.T., Roberts, M.A., & Rhoades, E.R., *Relative resistance to penicillin in the pneumococcus: A prevalence and case–control study.* Journal of the American Medical Association, 1980. **243**(18): p. 1824–1827.

202. Samore, M.H., Magill, M.K., Alder, S.C., Severina, E., Morrison-de Boer, L., Lynn Lyon, J., Carroll, K., Leary, J., Bishop Stone, M., Bradford, D., Reading, J., Tomasz, A., & Sande, M., *High rates of multiple antibiotic resistance in Streptococcus pneumoniae from healthy children living in isolated rural communities: Association with cephalosporin use and intrafamilial transmission.* Pediatrics 2001. **108**(4): p. 856–865.

203. Samore, M.H., Lipsitch, M., Alder, S.C., Haddadin, B., Stoddard, G., Williamson, J., Sebastian, K., Carroll, K., Ergonul, O., Carmeli, Y., & Sande, M.A., *Mechanisms by which antibiotics promote dissemination of resistant pneumococci in human populations.* American Journal of Epidemiology, 2005. **163**(2): p. 160–170.

204. Sattler, C.A., Mason, E.O., & Kaplan, S.L., *Prospective comparison of risk factors and demographic and clinical characterisitcs of community-acquired, methicillin-resistant versus methcillin-susceptible Staphylococcus aureus infection in children.* Pediatric Infectious Disease Journal, 2002. **21**(10): p. 910–916.

205. Sax, H., Harbarth, S., Gavazzi, G., Henry, N., Schrenzel, J., Rohner, P., Michel, J.P., & Pittet, D., *Prevalence and prediction of previously unknown MRSA carriage on admission to a geriatric hospital.* Age and Ageing, 2005. **34**(5): p. 456–462.

206. Schneider-Lindner, V., Delaney, J.A., Dial, S., Dascal, A., & Suissa, S., *Antimicrobial drugs and community-acquired methicillin-resistant Staphylococcus aureus, United Kingdom.* Emerging Infectious Diseases, 2007. **13**(7): p. 994–1000.

207. Schrag, S.J., Pena, C., Fernandez, J., Sanchez, J., Gomez, V., Perez, E., Feris, J.M. & Besser, R.E., *Effect of short-course, high-dose amoxicillin therapy on resistant pneumococcal carriage: A randomized trial.* Journal of the American Medical Association, 2001. **286**(1): p. 49–56.

208. Seaton, R.A., Steinke, D. T., Philipps, G., MacDonald, T., & Davey, P.G., *Community antibiotic therapy, hospitalization and subsequent respiratory tract isolation of Haemophilus influenzae resistant to amoxycillin: a nested case–control study.* Journal of Antimicrobial Chemotherapy, 2000. **46**(2): p. 307–309.

209. Seppala, H., Klaukka, T., Lehtonen, R., Nenonen, E., the Finnish Study Group for Antimicrobial Resistance & Huovinen, P., *Outpatient use of erythromycin: Link to increase erythromycin resistance in Group A streprococci.* Clinical Infectious Diseases, 1995. **21**(6): p. 1378–1385.

210. Skiest, D.J., Brown, K., Cooper, T.W., Hoffman-Roberts, H., Mussa, H.R., & Elliott, A.C., *Prospective comparison of methicillin-susceptible and methicillin-resistant community-associated Staphylococcus aureus infections in hospitalized patients.* Journal of Infection, 2007. **54**(5): p. 427–434.

211. Skull, S.A., Shelby-James, T., Morris, P.S., Perez, G., Yonovitz, A., Krause, V., Roberts, L.A., & Leach, A.J., *Streptococcus pneumoniae antibiotic resistance in Northern Territory children in day care.* Journal of Paediatrics and Child Health, 1999. **35**(5): p. 466–471.

212. Sotto, A., DeBoever, C.M., Fabbro-Peray, P., Gouby, A., Sirot, D., & Jourdan, J., *Risk factors for antibiotic-resistant Escherichia coli isolated from hospitalized patients with urinary tract infections: a prospective study.* Journal of Clinical Microbiology, 2001. **39**(2): p. 438–444.

213. Sportel, J.H., Koeter, G.H., van Altena, R., Lowenberg, A., & Boersma, W.G., *Relation between beta-lactamase producing bacteria and patient characteristics in chronic obstructive pulmonary disease (COPD)* Thorax, 1995. **50**: p. 249–253.

214. Steinke, D.T., Seaton, R.A., Philipps, G., MacDonald, T.M. & Davey, P.G., *Factors associated with trimethoprim-resistant bacteria isolated from urine samples.* Journal of Antimicrobial Chemotherapy, 1999. **43**(6): p. 841–843.

215. Steinke, D.T., Seaton, R.A., Phillips, G., MacDonald, T.M., & Davey, P., *Prior trimethoprim use and trimethoprim-resistant urinary tract infection: a nested case–control study with multivariate analysis and for other risk factors.* Journal of Antimicrobial Chemotherapy, 2001. **47**(6): p. 781–787.

216. Sundqvist, M., *Antibiotic resistance and population dynamics of Escherichia coli in relation to a large scale antibiotic consumption intervention*, in *Medicine*. 2010, Uppsala University: Uppsala, Sweden. p. 78.

217. Syrogiannopoulos, G.A., Grivea, I.N., Davies, T.A., Katopodis, G.D., Appelbaum, P.C., & Beratis, N.G., *Antimicrobial use and colonization with erythromycin-resistant Streptococcus pneumoniae in Greece during the first 2 years of life.* Clinical Infectious Diseases, 2000. **31**: p. 887–893.

218. Syrogiannopoulos, G.A., Katopodis, G.D., Grivea, I.N., & Beratis, N.G., *Antimicrobial use and serotype distribution of nasopharyngeal Streptococcus pneumoniae isolates recovered from Greek children younger than 2 years old.* Clinical Infectious Diseases, 2002. **35**(10): p. 1174–1182.

219. Tacconelli, E., De Angelis, G., Cataldo, M.A., Pozzi, E. & Cauda, R., *Does antibiotic exposure increase the risk of methicillin-resistant Staphlococcus aureus (MRSA) isolation? A systematic review and meta-analysis.* Journal of Antimicrobial Chemotherapy, 2008. **61**(1): p. 26–38.

220. Talan, D.A., Krishnadasan, A., Abrahamian, F.M., Stamm, W.E., & Moran, G.J., *Prevalence and risk factor analysis of trimethoprim-sulfamethoxazole-and fluoroquinolone-resistant Escherichia coli infection among emergency department patients with pyelonephritis* Clinical Infectious Diseases, 2008. **47**(9): p. 1150–1161.

221. Tentolouris, N., Jude, E.B., Smirnof, I., Knowles, E.A., & Boulton, A.J.M., *Methicillin-resistant Staphylococcus aureus: an increasing problem in a diabetic foot clinic.* Diabetic Medicine, 1999. **16**(9): p. 767–771.

222. Tentolouris, N., Petrikkos, G., Vallianou, N., Zachos, C., Daikos, G.L., Tsapogas, P., Markou, G., & Katsilambros, N., *Prevalence of methicillin-resistant Staphylococcus aureus in infected and uninfected diabetic foot ulcers.* Clinical Microbiology and Infection, 2006. **12**(2): p. 186–189.

223. Toltzis, P., Dul, M., O'Riordan, M.A., Toltzis, H., & Blumer, J.L., *Impact of amoxicillin on pneumococcal colonization compared with other therapies for acute otitis media.* The Pediatric Infectious Disease Journal, 2005. **24**(1): p. 24–28.

224. Toltzis, P., Dul, M., O'Riordan, M.A., Toltzis, H. & Blumer, J.L., *Comparitive effects of single-dose ceftriaxone versus three oral antibiotic regimens on stool colonization by resistant bacilli in children.* The Pediatric Infectious Disease Journal, 2007. **26**(1): p. 25–30.

225. Tomasson, G., Gudnason, T., & Kristinsson, K.G., *Dynamics of pneumococcal carriage among healthy Icelandic children attending day-care centres.* Scandinavian Journal of Infectious Diseases, 2005. **37**(6–7): p. 422–428.

226. Troillet, N., Carmeli, Y., Samore, M.H., Dakos, J., Eichelberger, K., DeGirolami, P.C. & Karchmer, A.W., *Carriage of methicillin-resistant Staphylococcus aureus at hospital admission.* Infection Control and Hospital Epidemiology, 1998. **19**(3): p. 181–185.

227. Tsolia, M., Kouppari, G., Zaphiropuulou, A., Gavrili, S., Tsirepa, M., Kafetzis, D., & Karpathhios, T., *Prevalence and patterns of resistance of Streptococcus pneumoniae strains isolated from carriers attending day care centers in the area of Athens.* Microbial Drug Resistance, 1999. **5**(4): p. 271–278.

228. Urbanek, K., Kolar, M., Strojil, J., Koukalova, D., Cekanova, L., & Hejnar, P., *Utilization of fluoroquinolones and Escherichia coli resistance in urinary tract infection: inpatients and outpatients.* Pharmacoepidemiology and Drug Safety, 2005. **14**(10): p. 741–745.

229. Ussery, X.T., Gessner, B.D., Lipman, H., Elliot, J.A., Crain, M.J., Tien, P.C., Parkinson, A.J., Davidson, M. Facklam, R.R., & Breiman, R.F., *Risk factors for nasopharyngeal carriage of resistant Streptococcus pneumoniae and detection of a multiply resistant clone among children living in the Yukon-Kuskokwim Delta region of Alaska.* The Pediatric Infectious Disease Journal, 1996. **15**(11): p. 986–992.

230. van de Sande-Bruinsma, N., Grundmann, H., Verloo, D., Tiemersma, E., Monen, J., Goossens, H, Ferech, M., & the European Antimicrobial Resistance Surveillance System & European Surveillance of Antimicrobial Consumption Project Groups, *Antimicrobial drug use and resistance in Europe.* Emerging Infectious Diseases, 2008. **14**(11): p. 1722–1730.

231. Van der Veen, E.L., Schilder, A.G.M., Timmers, T.K., Rovers, M.M., Fluit, A.C., Bonten, M.J., & Leverstein-van Hall, M.A., *Effect of long-term trimethoprim/sulfamethoxazole treatment on resistance and integron prevalence in the intestinal flora: a randomized, double-blind, placebo-controlled trial in children.* Journal of Antimicrobial Chemotherapy, 2009. **63**(5): p. 1011–1016.

232. Van Eldere, J., Mera, R.M., Miller, L.A., Poupard, J.A., & Amrine-Madsen, H., *Risk factors for development of multiple-class resistance to Streptococcus pneumoniae strains in Belgium over a 10-year period: Antimicrobial consumption, population density, and geographic location.* Antimicrobial Agents and Chemotherapy, 2007. **51**(10): p. 3491–3497.

233. Vanderkooi, O.G., Low, D.E., Green, K., Powis, J.E. & McGeer, A for the Toronto Invasive Bacterial Disease Network, *Predicting antimicrobial resistance in invasive pneumococcal infections.* Clinical Infectious Diseases, 2005. **40**(9): p. 1288–1300.

234. Vasquez, G.A., Siu, H.R., Luna, E.M., Reyes, K.C., & Zervos, M.J., *Risk factors for quinolone-resistant Escherichia coli urinary tract infection.* Infectious Diseases in Clinical Practice, 2009. **17**(5): p. 309–313.

235. Vellinga, A., Murphy, A.W., Hanahoe, B., Bennett, K., & Cormican, M., *A multilevel analysis of trimethoprim and ciprofloxacin prescribing and resistance of uropathogenic Escherichia coli in general practice.* Journal of Antimicrobial Chemotherapy, 2010. **65**(7): p. 1514–1520.

236. Volonakis, K., Souli, M., Kapaskelis, A., Bazaika, F., Grammelis, V., Ziakas, P.D., & Giamarellou, H., *Evolution of resistance patterns and identification of risk factors for Streptococcus pneumoniae colonization in daycare centre attendees in Athens. Greece.* International Journal of Antimicrobial Agents, 2006. **28**(4): p. 297–301.

237. Weber, P., Plaisance, J., & Mancy, C., *Lack of increase in resistance to quinolones in general practice isolates of Escherichia coli.* Journal of Antimicrobial Chemotherapy, 1994. **34**(1): p. 187–188.

238. Welby, P.L., Keller, D.S., Cromien, J.L., Tebas, P., & Storch, G.A., *Resistance to pencillin and non-beta-lactam antibiotics of Streptococcus pneumoniae at a children's hospital.* The Pediatric Infectious Disease Journal, 1994. **13**(4): p. 281–287.

239. Wright, S.W., Wrenn, K.D., Haynes, M.L., *Trimethoprimn-sulfamethoxazole resistance among urinary coliform isolates.* Journal of General Internal Medicine, 1999. **14**(10): p. 606–609.

240. Wright, S.W., Wrenn, K.D., Haynes, M.L., & Haas, D.W., *Prevalence and risk factors for multidrug resistant uropathogens in ED patients.* American Journal of Emergency Medicine, 2000. **18**(2): p. 143–146.

241. Yagci, D., Yoruk, F., Azap, A, & Memikoglu, O., *Prevalence and risk factors for selection of quinolone-resistant Escherichia coli strains in fecal flora of patients receiving quinolone therapy.* Antimicrobial Agents and Chemotherapy, 2009. **53**(3): p. 1287–1289.

242. Yagupsky, P., Porat, N., Fraser, D., Prajgrod, F., Merires, M., McGee, L., Klugman, K.P., & Dagan, R., *Acquisition, carriage, and transmission of pneumococci with decreased antibiotic susceptibility in young children attending a day care facility in southern Israel. .* The Journal of Infectious Diseases, 1998. **177**(4): p. 1003–1012.

243. Zaidi, M.B., Zamora, E., Diaz, P., Tollefson, L., Fedorka-Cray, P.J., & Headrick, M.L., *Risk factors for fecal quinolone-resistant Escherichia coli in Mexican children.* Antimicrobial Agents and Chemotherapy, 2003. **47**(6): p. 1999–2001.

## Competing interests

The authors declare that they have no competing interests.

## Authors’ contribution

BB performed the searches, analysed the data, and drafted first sections of the text. MP designed the study and formulated the hypotheses. FS led the APRES team. HG and ES provided their expertise in microbiology. MP and BB are the guarantors. All authors had full access to all of the data (including statistical reports and tables) in the study and can take responsibility for the integrity of the data and the accuracy of the data analysis. All authors read and approved the final manuscript.

## Pre-publication history

The pre-publication history for this paper can be accessed here:

http://www.biomedcentral.com/1471-2334/14/13/prepub

## Data sharing

Limited to other researchers on personal request.
